# Consequences of iron exposure and glutathione depletion on redox balance, lipidome, and neurotransmission in *C. elegans*

**DOI:** 10.1016/j.redox.2026.104023

**Published:** 2026-01-12

**Authors:** Anna Gremme, Emely Gerisch, Dominik Wieland, Julia Hillebrand, Franziska Drews, Marcello Pirritano, Ann-Kathrin Weishaupt, Janina Fuss, Vera Schwantes, Johannes Scholz, Vivien Michaelis, Alicia Thiel, Gawain McColl, Bernhard Michalke, Martin Simon, Heiko Hayen, Julia Bornhorst

**Affiliations:** a Food Chemistry with Focus on Toxicology, Faculty of Mathematics and Natural Science, University of Wuppertal, Wuppertal, 42119, Germany; b Institute of Inorganic and Analytical Chemistry, University of Münster, Münster, 48149, Germany; c Molecular Cell Biology and Microbiology, Faculty of Mathematics and Natural Science, University of Wuppertal, Wuppertal, 42119, Germany; d Competence Centre for Genomic Analysis, CCGA, Kiel, 24118, Germany; e Florey Institute of Neuroscience and Mental Health, The University of Melbourne, Parkville, Victoria, 3052, Australia; f Chemconsulting, Markt Schwaben, 85570, Germany; g TraceAge - DFG Research Unit on Interactions of Essential Trace Elements on Healthy and Diseased Elderly (FOR 2558), Berlin, Potsdam, Jena, Wuppertal, Germany

**Keywords:** Iron, Glutathione, Mitochondria, Lipids, Neurotransmitters, *C. elegans*

## Abstract

Although the redox active essential trace element iron (Fe) is involved in many important biological processes, an overexposure can lead to the excessive formation of reactive oxygen and nitrogen species (RONS). Thus, total Fe accumulation, as for example observed in neurodegenerative diseases or diseases as hemochromatosis, can lead to adverse consequences, especially if the antioxidant system is weakened. This system, and especially the most abundant antioxidant in organisms, glutathione (GSH), can be impaired by excess RONS levels, which is relevant during aging and in the context of neurodegenerative diseases. In this study, we demonstrate the consequences of Fe overdosing or/and GSH depletion in *Caenorhabditis elegans* (*C. elegans*) on Fe homeostasis, mitochondrial mass, phospho- and sphingolipidome, and on the neurotransmitter levels of acetylcholine, serotonin, dopamine, and γ-aminobutyric acid. In order to investigate this, we treated L4 nematodes with Fe(III) ammonium citrate (FAC) for 24 h or/and diethyl maleate (DEM) for 2 h or 24 h. While FAC treatment alone did not affect mitochondrial mass and cardiolipin content, it increased the amount of several lipid classes and the neurotransmitter acetylcholine. Treatment with DEM alone resulted in GSH depletion by 70 % and was associated with decreased mitochondrial mass and increased Fe(II), lipid, acetylcholine, and serotonin levels. Genes involved in GSH biosynthesis, Fe homeostasis, mitochondrial stress response, lipid biosynthesis, and neurotransmitter regulation are differentially expressed after DEM treatment. In addition, we were able to determine the GSH-DEM product in the nematode using HPLC-MS/MS. Although FAC treatment increased total Fe content in the nematode fivefold, the combined treatment with DEM showed no further effects compared to treatment with FAC or DEM alone. Together, these findings highlight the consequences of an impaired intracellular redox system on mitochondria, lipidome, and neurological endpoints, and identify several pathways, metabolites, and potential compensatory as well as long lasting effects.

## Highlights

• Iron supplementation affected phospho- and sphingolipidome, and acetylcholine level.
• Glutathione-diethyl maleate could be measured via HPLC-MS/MS.
• Diethyl maleate depleted glutathione levels, increased Fe(II), and affected genes associated with Fe homeostasis.
• Diethyl maleate impaired the mitochondria and upregulated the stress response gene *hsp-6*/*hspa-9*.
• Diethyl maleate affected phospho- and sphingolipid, serotonin, and acetylcholine metabolism.

## Graphical abstract

**Figure:**
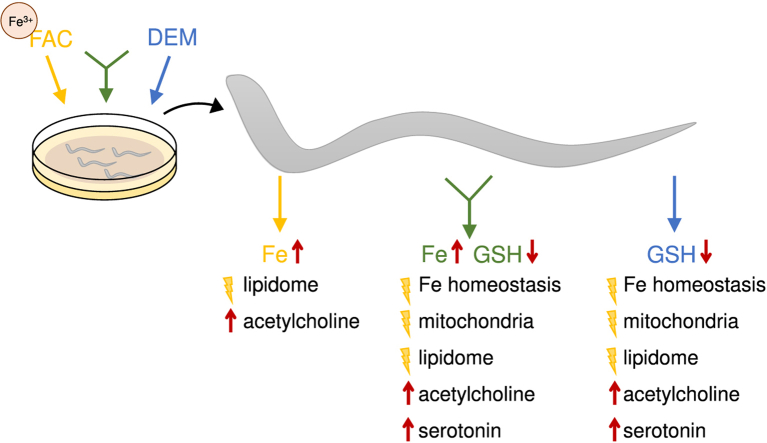

## 1 Introduction

Iron (Fe), an essential trace element, is involved in many cellular processes, including energy production, DNA synthesis, and oxygen transport [1,2]. These functions rely on the continuous redox cycling between Fe(II) and Fe(III), which may in an uncontrolled manner lead to adverse consequences such as the enhanced formation of reactive oxygen and nitrogen species (RONS) via the Fenton reaction [3]. Higher levels of RONS increase the risk of adverse modifications to biomolecules such as lipids, proteins, and DNA [4]. Therefore a particular perturbation of cellular function and integrity arises if Fe levels are increased, as it has been observed in aging human brains and diseases like hemochromatosis or diabetes mellitus [5]. Total Fe accumulation in the brain is further associated with cognitive impairments and even neurodegenerative diseases such as Alzheimer's (AD) and Parkinson's diseases, but the underlying mechanisms are not fully understood [5,6]. Due to health risks associated with excessive Fe intake from food and food supplements, the European Food Safety Authority (EFSA) has defined safe intake levels of 10 mg/day for children and 40 mg/day for adults in 2024 [7]. Under optimal conditions, cells possess a robust antioxidative system to prevent excessive RONS production and oxidative stress. However, antioxidative capacity can be depleted by enhanced RONS production, toxins, or by aging, which can lead, among other effects, to a deficiency of the important antioxidant glutathione (GSH) [8]. This endogenous tripeptide is one of the most abundant intracellular antioxidants, as it functions as a radical scavenger and as a co-enzyme for several enzymes like glutathione peroxidases (GPX), glutaredoxins, and glutathione-S-transferases (GST) [9]. As a co-factor of GPX, GSH plays a crucial role in lipid hydroperoxides reduction and is therefore considered an important antioxidant in the inhibition of Fe-dependent cell death, ferroptosis [10]. Additionally, GSH plays an important role in Fe homeostasis, as it forms the major complex of the labile Fe pool (LIP). Thereby free Fe is prevented from accumulating while ensuring its rapid availability, for example, for incorporation into Fe-dependent enzymes [11]. The biosynthesis of heme and Fe-sulfur clusters primarily takes place in the mitochondria, leading to higher amounts of Fe in this organelle [12]. This, combined with the high production of superoxide radicals during oxidative phosphorylation, increases the potential for mitochondrial oxidative stress and requires enhanced antioxidant defense [13]. Since neurons have a high energy demand, they are rich in mitochondria, and impairments of these organelles are often discussed in the context of neuronal dysfunction and neurodegenerative diseases [14]. This underlines the importance of a balanced Fe homeostasis and cellular redox system.

This raises the question of the consequences for an organism under conditions of Fe overexposure and GSH depletion. To gain further mechanistic insights, we established a model organism exhibiting increased Fe amounts, decreased GSH levels, or a combination of both. For this purpose, we treated the nematode *Caenorhabditis elegans* (*C. elegans*) with iron(III) ammonium citrate (FAC) or/and the GSH-binding molecule diethyl maleate (DEM) [15,16]. In this study, L4 worms were treated with FAC for 24 h, followed by short-term (2 h) and long-term (24 h) treatment with DEM. These two time points resemble acute or long-term effects, with the latter being more relevant for adverse effects from chronic exposure.

The nematode *C. elegans* is a well-established model for investigating oxidative stress, redox homeostasis, and neurotoxicity. Owing to its favorable characteristics, including a fully sequenced genome, a rapid life cycle, and since it is a complete, multicellular organism, *C. elegans* is well aligned with the 3R principle (refine, reduce, replace). Moreover, it is widely used in toxicological and iron homeostasis research, as it shares numerous orthologous genes with mammals, including genes involved in oxidative stress responses, cell death pathways, neurological functions, and iron metabolism [[16], [17], [18]].

## 2 Materials and methods

### 2.1 *C. elegans* handling and treatment with FAC and DEM

The *C. elegans* strains Bristol N2 (wild type) and OH7193 (*him-8* (*e1489*)) were obtained from the *Caenorhabditis* Genetics Center (CGC).

The worms were cultivated on 8P agar plates coated with NA22 *E. coli* at 20 °C, as described previously. To ensure all experiments were performed using worms at larval stage 4 (L4), the nematodes were synchronized following established methods and subsequently grown on nematode growth medium (NGM) coated with OP50 *E. coli* [19,20]. L4 worms were treated for 24 h with FAC (reagent grade, Sigma Aldrich), which was freshly dissolved in bidistilled water and added to inactive *E. coli* on NGM agar plates before each experiment. Since the chemical formula of FAC (xFe ∙ yNH_4_C_6_H_7_O_8_) is semi defined, we determined a Fe content of 14.8 ± 0.3 % by weight (n = 3) using inductively coupled plasma-optical emission spectrometry (ICP-OES) (instrumental parameters in 2.3) in the FAC batch used. The 20 mM FAC used in this study corresponds to 5.3 mg/mL FAC. The *E. coli* was heat-inactivated as described previously [17]. Following 24 h FAC treatment, day 1 adult nematodes were treated with various concentrations of DEM (Sigma Aldrich) for either 2 h in 85 mM NaCl solution or for 24 h on NGM plates prepared in the same manner as for FAC treatment. DEM was diluted in DMSO, and the final concentration of DMSO was adjusted to 1 % v/v under all conditions. DEM treatment did not involve additional FAC exposure.

### 2.2 Protein determination via BCA assay

To normalize metal content, GSH, GSSG, nucleotides, lipids, malondialdehyde (MDA), and neurotransmitter levels to protein content, the protein amount was determined using bicinchoninic acid (BCA) assay as described previously [18].

### 2.3 Quantification of total Fe and Zn via ICP-OES

Quantification of total Fe and Zn was performed using ICP-OES (Avio 220 Max, PerkinElmer) as previously described [17]. The instrument parameters were as follows: Plasma power: 1500 W, cooling gas: 8 L/min, auxiliary gas: 0.2 L/min, nebulizer (MicroMist™) gas: 0.7 L/min. The analysis was performed using Yttrium (Y) as internal standard and the following element lines: Fe – 259.939 nm, Zn – 206.200 nm, Y – 371.029 nm. The element contents were normalized to protein amount.

### 2.4 Determination of GSH, GSSG, and GSH-DEM levels via HPLC-MS/MS

Quantification of reduced (GSH) and oxidized (GSSG) glutathione levels was performed using high performance liquid chromatography-tandem mass spectrometry (HPLC-MS/MS, Agilent 1290 Infinity II, Sciex QTrap 6500+ triple quadrupole MS) as described by Thiel et al. [21]. The product S-(α,β-bis(ethoxycarbonyl)ethyl)glutathione (GSH-DEM) of the reaction of GSH with DEM was previously characterized by Kubal et al. using ^1^H NMR analysis [15]. We adapted the method of Thiel et al. to include the determination of this molecule, with specific parameters detailed in the supplementary table (S1). GSH and GSSG were quantified using external calibration and normalized to protein content. To estimate the formation of the GSH-DEM product, the GSH-DEM/GSH ratio was calculated based on the integrated peak areas.

### 2.5 Transcriptomic analysis

For transcriptomic analysis, RNA was isolated from 750 day 1 and day 2 worms in 1 mL TRIreagent® (Sigma-Aldrich) using the phenol/chloroform extraction method as previously described [22]. Isolated RNA was purified with the RNA clean and concentrator™-5 Kit (Zymo Research) according to the manufacturer's instructions. The integrity of the RNA was verified using gel electrophoresis. Library preparation was performed with 300 ng RNA using the NEBNext® Ultra™ II directional RNA library prep kit for Ilumina® in conjunction with Poly(A) mRNA magnetic isolation (#E7760S, #E7490S, New England Biolabs) with 14 PCR cycles according to manufacturer's instructions.

Library concentration was verified using the Qubit™ 1X dsDNA HS Assay Kit (#Q33230, Thermo Fisher Scientific) and the Qsep1 Bio-Fragment Analyzer (BiOptic Inc.) with a Standard DNA Cartridge Kit (catalogue no.: C105201) to check the library for adapter contamination and size distribution.

Libraries were pooled and sequenced using a S4 Flowcell on the NovaSeq 6000 Illumina platform (Illumina) in 100 bp paired-end sequencing mode. De-multiplexing, quality trimming and adapter removal was performed using TrimGalore (version 0.6.5, www.bioinformatics.babraham.ac.uk) which uses cutadapt [23]. Reads were randomly subsampled using seqtk (https://github.com/lh3/seqtk) and single-end reads were mapped onto the *C. elegans* Bristol N2 genome using the bowtie2 plugin for Geneious Prime version 2025.1.2. Expression values of annotated genes were calculated in Geneious Prime. Differences between the sample groups were visualized by PCA based on transcripts per million (TPM) expression level data using the prcomp and ggbiplot visualization function in R. Differentially expressed genes (DEGs) were calculated using DESeq2 with a threshold of p-value <0.01 and log_2_ fold change >1/<-1 for DEG characterization [24]. The p-values were attained by the Wald test and corrected for multiple testing using the Benjamini and Hochberg method. Term analysis of DEGs was performed using the geneontology.org website [25,26] which uses PANTHER [27]. For visualization, an R-script from Ref. [28] was used. All sequencing data are available in the ArrayExpress database (http://www.ebi.ac.uk/arrayexpress) under accession number E-MTAB-15180.

### 2.6 Determination of survival rate

Survival rate was assessed to evaluate the lethality of DEM and combined FAC and DEM treatment. For this purpose, 20–30 worms were transferred to a 3.5 cm NGM agar plate coated with OP50 *E. coli* following 24 h FAC and 2 h DEM treatment. 24 h post DEM treatment, the percentage of adult worms still moving and thus surviving was determined.

### 2.7 Determination of labile Fe(II) via FerroOrange dye

To assess the cytosolic labile Fe(II) status after FAC and DEM treatment, 2250 day 1 and day 2 adult worms were treated with the BioTracker FerroOrange dye (Sigma-Aldrich). Following FAC and DEM treatment, the nematodes were washed three times with NaCl solution (85 mM, 0.01 % Tween® 20) to remove eggs and L1 worms by allowing the adult worms to sink to the bottom of the tube and removing the supernatant. The FerroOrange dye was freshly dissolved in DMSO just before each experiment, and the worms were treated with a final concentration of 10 μM dye for 2 h in the dark with gentle shaking. The worms were washed again three times with NaCl solution and transferred to NGM plates coated with OP50 *E. coli* for 1 h in the dark to excrete the dye from the intestine. After repeated washing and removal of the *E. coli*, the fluorescence of the dye (excitation: 540 nm, emission: 575 nm) and the auto-fluorescence of the worms (excitation: 405 nm, emission: 455 nm) for normalization were measured with a microplate reader (Infinite M Plex, Tecan). The scan was performed in a 3 × 3 filled square with multiple reads.

### 2.8 Gene expression via Taqman real-time (RT) qPCR analysis

Gene expression was measured using TaqMan gene expression assay probes (Applied Biosystems, Thermo Fisher Scientific) and a CFX duet RT PCR system (Bio-Rad) [29]. RNA isolation was performed as described in 2.5, and 1 μg of the isolated RNA was transcribed using the High-Capacity cDNA Reverse Transcription Kit (Applied Biosystems, Thermo Fisher Scientific) according to the manufacturer's instructions. Following Taqman probes were used: *smf-2* (Ce02496629_g1), *smf-3* (Ce02461545_g1), *ftn-1* (Ce02477612_g1), *ftn-2* (Ce02415799_g1), *fpn-1.1* (Ce02414545_m1), *fpn-1.2* (Ce02471200_g1) [30]. For normalization, the gene expression of the house keeper gene *afd-1* (afadin (*AFDN*) orthologue) (Ce02414573_m1) was measured and the comparative 2^−ΔΔCt^ method was used for evaluation [31].

### 2.9 Determination of mitochondrial mass via MitoTracker™ green FM

The mitochondrial mass was determined using MitoTracker™ Green FM (Thermo Fisher Scientific) [32]. This assay was carried out in *Pdat-1::mCherry* + P*ttx-3::mCherry* worms. Therefore, OH7193 worms were outcrossed and treated with FAC and DEM. The dye was dissolved in DMSO at a final concentration of 2.5 mM, and aliquots were stored at −20 °C. After FAC and DEM treatment, the worms were treated with 1 μM MitoTracker™ Green for 1 h in the dark with gentle shaking. After the worms had been washed to remove the dye, they were transferred to NGM plates coated with OP50 *E. coli* to excrete the dye from the intestine for 1 h. The fluorescence of the MitoTracker™ dye (excitation: 485 nm, emission: 525 nm) and the red fluorescence of the worms (excitation: 560 nm, emission: 599 nm) were measured using a microplate reader (Infinite M Plex, Tecan). The scan was performed in a 3 × 3 filled square with multiple reads. The green fluorescence of the MitoTracker Green FM was normalized to the red fluorescence of the worms measured in each well. As a positive control, day 1 adult worms were treated for 1 h with 100 μM sodium azide (≥99.5 %, Sigma Aldrich) [33].

### 2.10 Quantification of energy-related nucleotides via HPLC-DAD

The energy-related nucleotides adenosine triphosphate (ATP), adenosine diphosphate (ADP), adenosine monophosphate (AMP), nicotinamide adenine dinucleotide phosphate (NADPH), and nicotinamide adenine dinucleotide (NADH, NAD^+^) were quantified using HPLC with a diode array detector (DAD) according to Bornhorst et al. [34]. Following treatment with FAC and DEM, 150 μL KOH (0.5 M) was added to 1500 day 1 and day 2 adult worms suspended in 100 μL NaCl solution (85 mM). Immediately afterwards, the samples were homogenized with zirconia beads in a bead ruptor (40 s, high, Biolab Products), and exactly 60 s after the addition of KOH, neutralized with 30 μL H_3_PO_4_ (10 %). After centrifugation (20 min, 18620 rcf, 4 °C), the nucleotides and the protein content were measured from the supernatant. Detection was carried out at 259 nm, and quantification was performed using an external calibration for each nucleotide. Sodium azide was used as positive control as described in 2.8. Nucleotide levels were normalized to protein content.

### 2.11 Determination of cardiolipins (CL) via 2D heart-cut HPLC-MS/MS

To determine CL, 4500 day 1 and day 2 adult worms were washed in 1 mL NaCl solution (85 mM, 0.01 % Tween® 20), and 50 μL of the suspension was taken for protein determination. The worm pellet was resuspended in 100 μL NaCl solution for lipid extraction. Prior to lipid extraction, according to a protocol based on Matyash et al., all samples underwent three freeze thaw cycles followed by sonication in an ultrasonic processor for cell disruption [35,36]. The detailed protocol is provided in the supporting information (S3).

For determination of CL distribution, 2D heart-cut HPLC-MS/MS (Vanquish Flex Duo UHPLC-system, Q Exactive Plus; Thermo Fisher Scientific) was performed (S5, S6). Chromatographic data analysis and CL identification were carried out with the open-source software MZmine 4 (version 4.2.0; mzio GmbH) (S9) [37]. To analyze CL distribution, relative peak areas of the identified CL species were determined (Fig. S5). The sums of CL were normalized to the internal standard CL 64:4 and the protein content. Absolute quantification was not performed.

### 2.12 Determination of the relative gene expression of mitochondrial expressed *nd-1* via RT-qPCR

The determination of the relative gene expression of mitochondrial expressed *nd-1* via RT-qPCR is based on the protocol by Rooney et al. [29]. RNA isolation from 750 adult worms and transcription were performed as described in 2.8. *Nd-1* (Primer: 5‘-ATTGCTGAACTTAACCGGGC-3‘, 3‘-GCTACTCTGGCAAACTCCAC-5‘) was chosen as mitochondrial expressed gene and *cox-4* (Primer: 5’-GCCGACTGGAAGAACTTGTC-3′, 3‘-GCGGAGATCACCTTCCAGTA-5‘), as the nuclear expressed gene, was used for normalization. The RT-qPCR was performed in 20 μL with the following final concentrations: SYBR Green: 1X, Primer: 400 nM, cDNA: 5 ng/μL with a CFX duet RT PCR system (Bio-Rad). 40 amplification cycles were performed and the PCR products were monitored by dissociation curve. The evaluation was carried out using the 2^−ΔΔCt^ method [31].

### 2.13 Determination of phospho- (PL) and sphingolipid (SL) composition

For determination of PL and SL compositions, worm pellets and lipid extraction were conducted as stated above (see 2.11). Mass spectrometric measurements, including ion mobility spectrometry, were performed on a timsTOF fleX (Bruker Daltonics) mass spectrometer after reversed phase-HPLC separation utilizing an UltiMate 3000 UHPLC system (Thermo Fisher Scientific). Data processing and evaluation was carried out with the Metaboscape 2023b software (Bruker Daltonics). Details regarding mass spectrometric and chromatographic methodologies are provided in the supporting information (S7, S8, S9).

### 2.14 Quantification of MDA via HPLC-FLD

MDA was quantified using HPLC with fluorescence detection (HPLC-FLD, Agilent 1260 Infinity II) according to Weishaupt et al. [38]. After treatment with FAC and DEM, 1500 day 1 and day 2 adult worms were pelletized and processed for analysis within one week. Results were normalized to protein content.

### 2.15 Quantification of neurotransmitter level via HPLC-MS/MS

The neurotransmitters acetylcholine, serotonin, dopamine, and γ-aminobutyric acid were quantified via HPLC-MS/MS (Agilent 1290 Infinity II, Sciex QTrap 6500+ triple quadrupole MS) as described previously by Weishaupt et al. [39]. For analysis, 1500 day 1 and day 2 adult worms were pelleted following FAC and DEM treatment, and neurotransmitters were quantified using deuterated internal standards [39]. The results were normalized to protein content.

### 2.16 Aldicarb sensitivity assay

To investigate the synaptic transmission of cholinergic neurons, the aldicarb-sensitivity assay according to Mahoney et al. was performed [40]. Aldicarb (Sigma-Aldrich) was dissolved in 70 % ethanol and added to NGM agar at a final concentration of 2 mM. The assay was performed as previously described, and the number of non-paralyzed worms was counted every 60 min for 360 min [39].

### 2.17 Statistical analysis

Statistical analysis was performed using GraphPad Prism 6 (GraphPad Software). T-test with α = 0.05 was used with Welch's correction and the following significance levels: ∗p ≤ 0.05, ∗∗p ≤ 0.01, ∗∗∗p ≤ 0.001 (for transcriptomic analysis, see 2.5).

## 3 Results

### 3.1 FAC increased the total Fe level and DEM bound to GSH

**Fig. 1:**
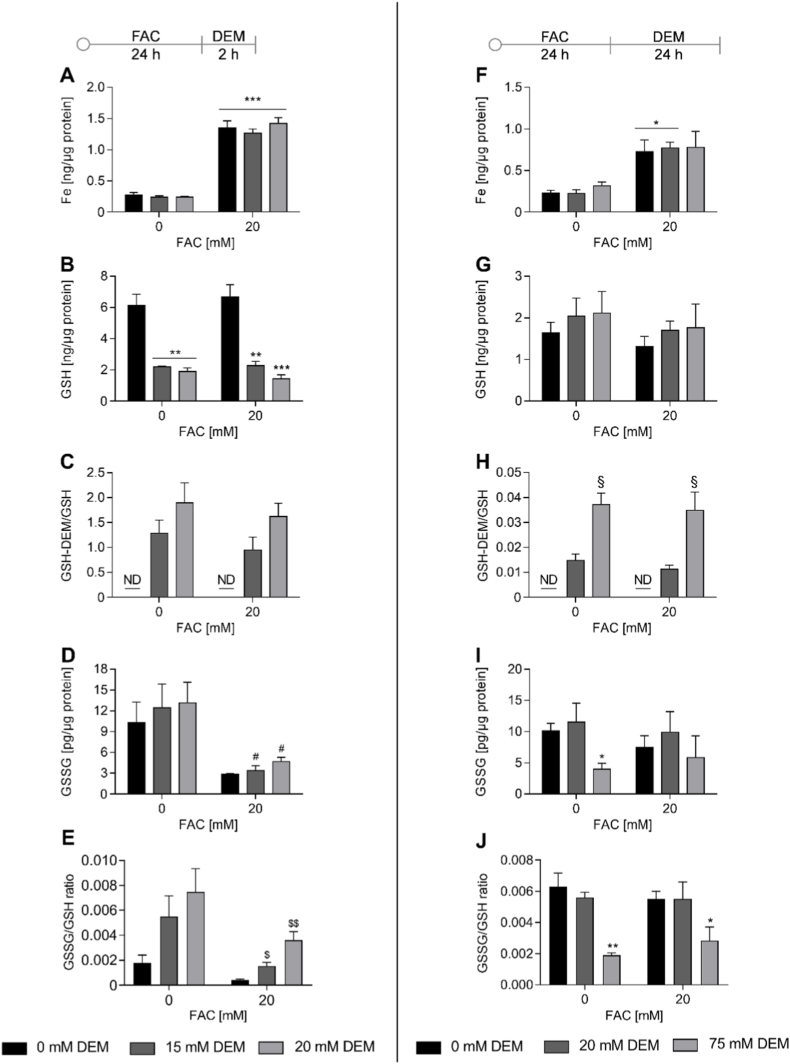
Increased Fe levels and glutathione status after treatment with FAC and 2 h (A–E) or 24 h (F–J) treatment with DEM. A, F) Total Fe was measured via ICP-OES. B, G) GSH levels, C, H) GSH-DEM/GSH ratio, and D, I) GSSG levels were measured via HPLC-MS/MS. E, J) GSSG/GSH. Total Fe, GSH, and GSSG levels were normalized to protein content and GSSG/GSH ratio was calculated from normalized GSSG and GSH values. Shown are the mean + SEM of ≥ 3 independent experiments. Significance is depicted as ∗ compared to untreated control, § compared to other DEM concentrations,# to DEM treatment only, and $ to FAC treatment only. ND = not detectable.

To verify the total Fe content of the worms after FAC and DEM treatment, Fe was determined using ICP-OES. 24 h treatment with 20 mM FAC increased the total Fe content in the nematodes fivefold (Fig. 1A) and even 24 h post Fe treatment, the Fe content was 3 times higher compared to untreated control (Fig. 1F). While Fe levels were increased, the Zn content was decreased at both time points (Fig. S3). DEM had no effect on the Fe content at any tested concentration after both treatment times. HPLC-MS/MS measurement revealed that the short-term treatment of 2 h with DEM decreased GSH level to 30 % (Fig. 1B), while the ratio of GSH-DEM to GSH was between 1 and 2 (Fig. 1C). Whereas DEM treatment alone had no impact on GSSG level, GSSG was markedly decreased after FAC treatment alone and combined with DEM (Fig. 1D). Although long-term treatment with DEM for 24 h showed no impact on total GSH levels (Fig. 1G), a ratio of 0.015 ± 0.002 and 0.037 ± 0.004 of GSH-DEM to GSH could be determined depending on the DEM concentration (Fig. 1H). In contrast to the short-term treatment, GSSG levels were decreased after 24 h treatment with 75 mM DEM (Fig. 1I). Based on this and the higher GSH-DEM to GSH ratio, solely 75 mM DEM was used in the investigation of further endpoints for the long-term treatment. The altered GSSG and GSH data led to decreased GSSG/GSH ratios after treatment with FAC alone and combined with 2 h DEM treatment (Fig. 1E). In addition, 24 h treatment with 75 mM DEM alone and combined with FAC treatment led to decreased GSSG/GSH ratios (Fig. 1J).

### 3.2 Transcriptomic analysis

**Fig. 2:**
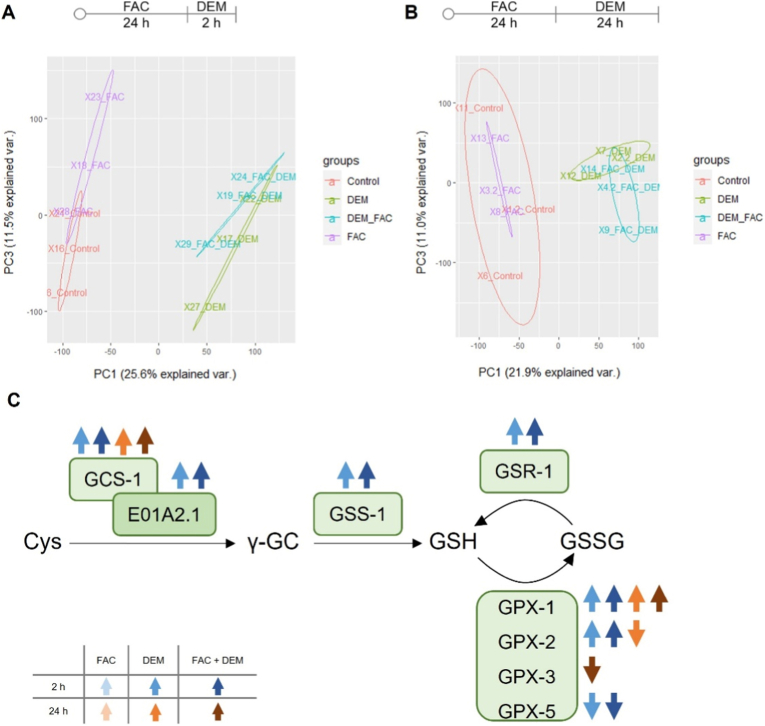
Principal component analysis (PCA) of PC1 and PC3 after treatment with FAC and 2 h (A) or 24 h (B) with DEM. C) Proteins encoded by DEGs of GSH metabolism. Transcriptomic analysis was performed from n = 3 independent experiments each. *C. elegans*/human orthologue: GCS-1/GCLC: glutamate-cysteine ligase; E01A2.1/GCLM: glutamate-cysteine ligase catalytic subunit; GSS-1/GSS: glutathione synthase; GSR-1/GSR: glutathione disulfide reductase; GPX-1/2/GPX-4: glutathione peroxidase; GPX-3/5/GPX-3: glutathione peroxidase.

Transcriptomic analysis was performed to get an overview of the mechanistic consequences at the level of gene expression after FAC or/and DEM treatment. The principal component analysis (PCA) shows variations in the gene expression pattern between the untreated controls and the samples treated with DEM, either alone or in combination with FAC, in both treatment scenarios (Fig. 2A and B). FAC-treated samples showed almost no variance compared to untreated control at both times measured (2 h or 24 h post FAC treatment). However, the combined treatment with FAC and DEM partially shows other differentially expressed genes (DEGs) compared to untreated control than the treatment with DEM alone. Only 74 % of the genes overlap after 2 h DEM treatment and 59 % after 24 h DEM treatment (Fig. S4).

**Fig. 3:**
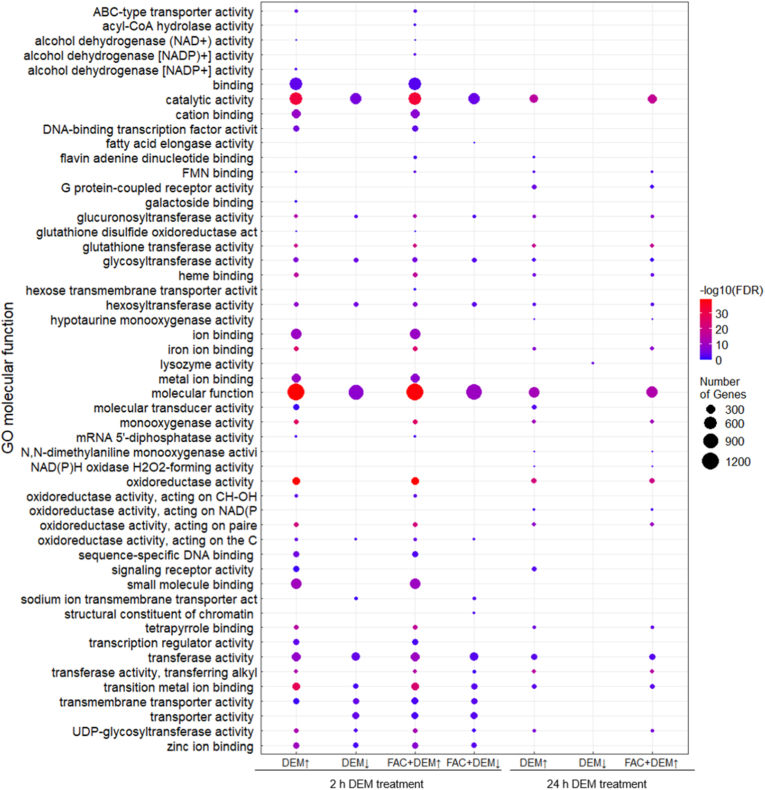
Heat map of a selection of Gene Ontology (GO) terms related to molecular function enriched among the up-(↑) and down-(↓) regulated DEGs after treatment with FAC and 2 h or 24 h DEM. No GO terms could be formed from the DEGs after treatment with FAC alone and the down-regulated genes after the combined treatment with FAC and 24 h DEM. Transcriptomic analysis was performed from n = 3 independent experiments each.

Transcriptomic analysis further indicates that DEM and the combined treatment with FAC led to changes in the expression of genes involved in GSH synthesis and GSH-dependent enzymes (Fig. 2C). In addition to the genes shown here, several GST genes were also differentially expressed after DEM treatment for 2 h and 24 h, which are represented by the Gene Ontology (GO) term ‘glutathione transferase activity’ (Fig. 3). Furthermore, several additional GO terms such as iron ion binding, catalytic activity, oxidoreductase activity, and molecular function were enriched among the DEGs, particularly after short-term treatment with DEM.

### 3.3 DEM affects survival rate, Fe redox status, and genes of Fe homeostasis

**Fig. 4:**
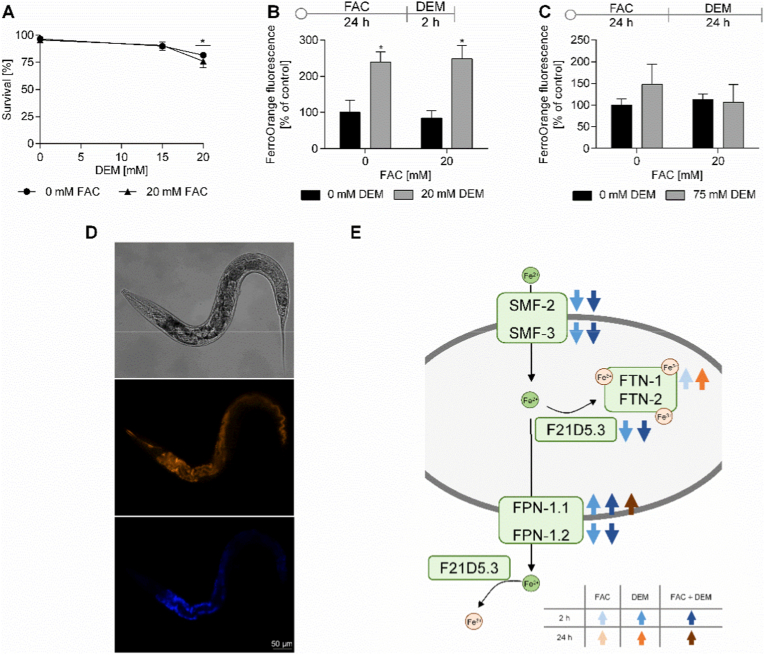
A) Survival rate after 24 h FAC and 2 h DEM treatment. B, C) Labile Fe(II) levels measured as FerroOrange fluorescence after treatment with FAC and 2 h (B) or 24 h (C) treatment with DEM were normalized to worm autofluorescence and untreated control. D) Representative bright field, FerroOrange fluorescence (orange), and auto fluorescence (blue, excitation: 405 nm, emission: 455 nm) images of wild type worms after treatment with FerroOrange dye. E) Proteins encoded by DEGs of Fe-homeostasis after treatment for 2 h and 24 h with FAC, DEM, and FAC + DEM compared to untreated control. Shown are the mean ∓ SEM of ≥ 3 independent experiments. Significance is depicted as ∗ compared to untreated control. Transcriptomic analysis was performed from n = 3 independent experiments each. *C. elegans*/human orthologue: SMF-2/3/DMT-1: divalent metal transporter; FTN-1/-2/FTH and FTL: ferritin; F21D5.3/DCYTB: duodenal cytochrome B; FPN-1.1/1.2/FPN: ferroportin.

To investigate the toxicity of FAC and DEM, the survival rate was examined 24 h after treatment. While short-term treatment with 15 mM DEM had no impact on the survival rate, 20 mM DEM alone led to a mortality of 15 % (Fig. 4A). Prior treatment with FAC for 24 h had no impact on this effect. Due to the slight toxicity of 20 mM DEM, this concentration was further used for short-term treatment. Long-term treatment with 20 mM and 75 mM DEM did not lead to a reduction in the survival rate (data not shown).

For the investigation of the Fe redox status, we treated the nematodes with FerroOrange following FAC or/and DEM treatment. This dye, also known as RhoNox-4, enables the detection of cytosolic labile Fe(II) through an irreversible reaction, resulting in a fluorescent compound (Fig. 4D) [41]. Even though the total Fe content increased markedly after FAC treatment, no alterations in the Fe(II) level could be observed by FAC only (Fig. 4B, C). However, the Fe(II) level increased significantly after short-term treatment with DEM both alone and combined with FAC to the same extent (Fig. 4B). Long-term treatment with DEM did not lead to any alterations (Fig. 4C).

**Fig. 5:**
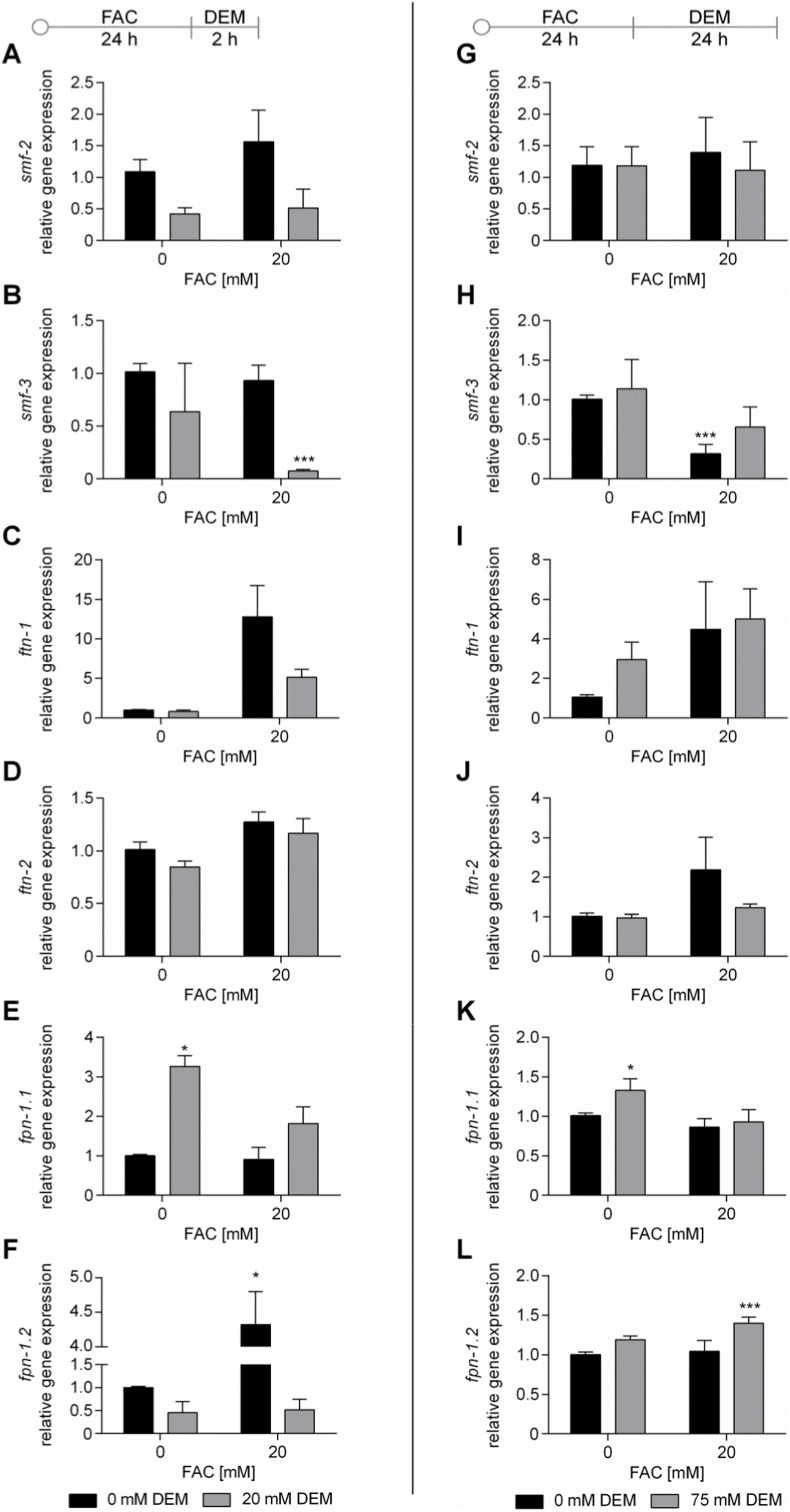
Relative gene expression after treatment with FAC or/and 2 h (A–F) or 24 h (G–L) treatment with DEM. Gene expression of A, G) *smf-2*; B, H) *smf-3*; C, I) *ftn-1*; D, J) *ftn-2*; E, K) *fpn-1.1* and F, L) *fpn-1.2* were determined via RT-qPCR. Shown are the mean + SEM of 3 independent experiments. Significance is depicted as ∗ compared to untreated control. *C. elegans*/human orthologue: SMF-2/3/DMT-1: divalent metal transporter; FTN-1/-2/FTH and FTL: ferritin; FPN-1.1/1.2/FPN: ferroportin.

Besides the redox status of Fe, changes in gene expression of Fe homeostasis genes also indicate an impact of DEM on Fe homeostasis (Fig. 4E). Transcriptomic analysis revealed the downregulation of the Fe-importers *smf-2* and *smf-3*, and of the ferroxidase *f21d5.3*. A trend toward decreased *smf-2* and *smf-3* gene expression after DEM alone and combined treatment with FAC was confirmed by RT-qPCR analysis, which also showed decreased gene expression levels of *smf-3* 24 h after FAC treatment (Fig. 5B, H). Interestingly, the Fe-exporter *fpn-1.1* is upregulated, while another exporter, *fpn-1.2*, is downregulated after 2 h treatment with DEM alone and combined with FAC. However, RT-qPCR analysis showed increased gene expression of *fpn-1.2* 24 h after treatment with FAC alone and combined with 24 h DEM (Fig. 5E, F, K, L). FAC alone and 24 h treatment with DEM led to increased gene expression of the Fe storage protein FTN-1, which stores Fe as Fe(III)-species; however, the effect was not significant (Figs. 4E and 5C, I).

### 3.4 DEM led to mitochondrial impairment

**Fig. 6:**
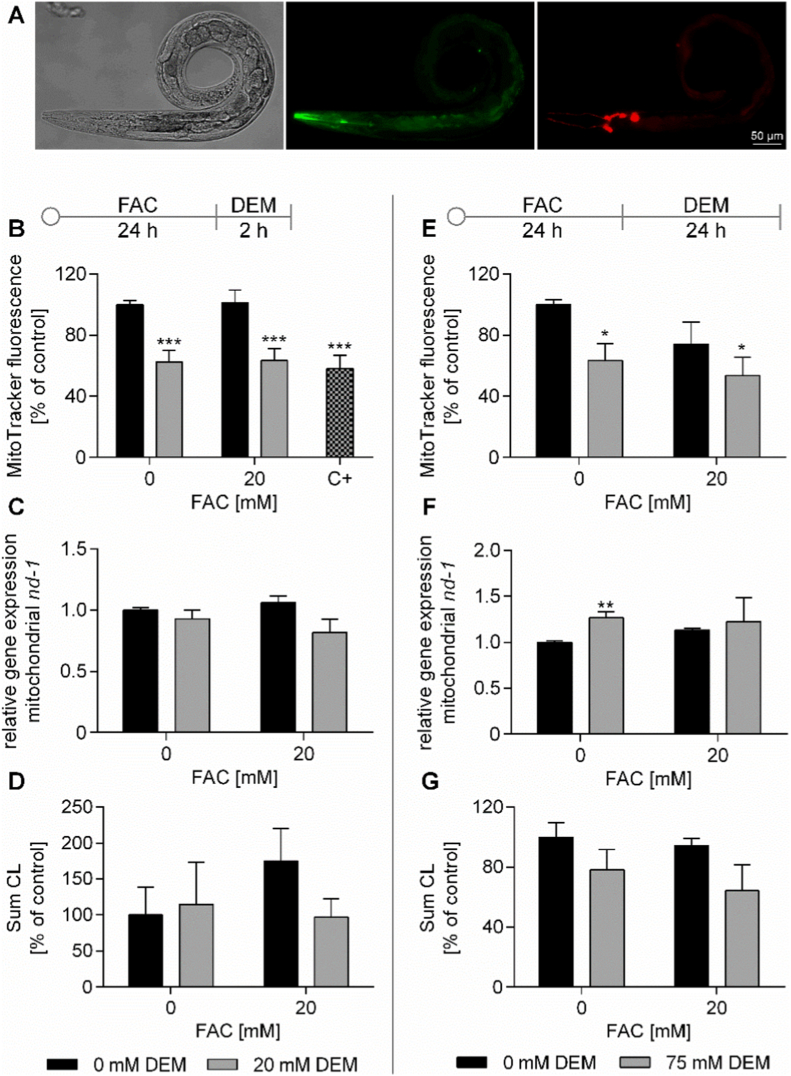
A) Representative bright field, MitoTracker™ Green FM (green), and mCherry fluorescence (red) images of P*dat-1::mCherry* + P*ttx-3::mCherry* worms after treatment with MitoTracker™ Green FM dye. MitoTracker fluorescence, relative gene expression of *nd-1* and the sum of cardiolipins (CL) were measured after treatment with FAC and 2 h (B, C, D) or 24 h (E, F, G) DEM. MitoTracker fluorescence (B, E) was normalized to mCherry fluorescence and untreated control. Relative gene expression of mitochondrial expressed *nd-1* (C, F) was determined using RT-qPCR and relative gene expression of mitochondrial *nd-1* was normalized to the relative gene expression of nuclear *cox-4* and control. CL (D, G) were determined via 2D heart-cut HPLC-MS/MS and normalized to internal standard (S3), protein content, and untreated control. Shown are the mean + SEM of ≥ 3 (CL: 2 h DEM n = 2) independent experiments. Significance is depicted as ∗ compared to untreated control. C+: 1 h treatment with 100 μM sodium azide as positive control.

We used the MitoTracker™ Green FM dye to determine the mitochondrial mass to investigate the impact of FAC or/and DEM on the mitochondrial number and activity. The fluorescence dye accumulated in the mitochondrial matrix in living worms, whereby the intensity of the fluorescence is evaluated as proportional to the mitochondrial mass (Fig. 6A) [42]. While treatment with FAC led to no alterations, DEM led to decreased mitochondrial masses alone and combined with FAC in both short-term and long-term treatments (Fig. 6B, E). DEM also affected the relative gene expression of *nd-1* (gene of the mitochondrial DNA), as short-term treatment showed a slight trend toward lower levels, whereas long-term treatment increased the expression (Fig. 6C, F). In addition, transcriptomic data revealed that short-term treatment with DEM alone and in combination with FAC resulted in an upregulation of *hsp-6*, which is involved in the mitochondrial stress response (Table 1) [43]. Although mitochondria are the main source of the energy nucleotide ATP, none of the treatments led to alterations in the overall cellular energy balance, calculated as energy charge value (AEC) (Fig. S5). The PL subclass cardiolipin (CL) was determined via 2D heart-cut HPLC-MS/MS. This class is exclusively located in the mitochondrial membrane and showed a trend toward reduced levels after 24 h DEM treatment, both alone and combined with Fe treatment (Fig. 6G). In addition, the distribution of CL was primarily affected by the 24 h treatment with DEM alone and in combination with FAC (Fig. S6).

**Table 1:** Upregulated gene expression of *hsp-6* after treatment with 2 h DEM, both alone and in combination with FAC. Transcriptomic analysis was performed from n = 3 independent experiments each.

	DEM (2 h)	FAC + DEM (2 h)	DEM (24 h)	FAC + DEM (24 h)
**stress response**	*hsp-6* ↑	*hsp-6* ↑	–	–

### 3.5 FAC and DEM treatment led to changes in the lipidome

**Fig. 7:**
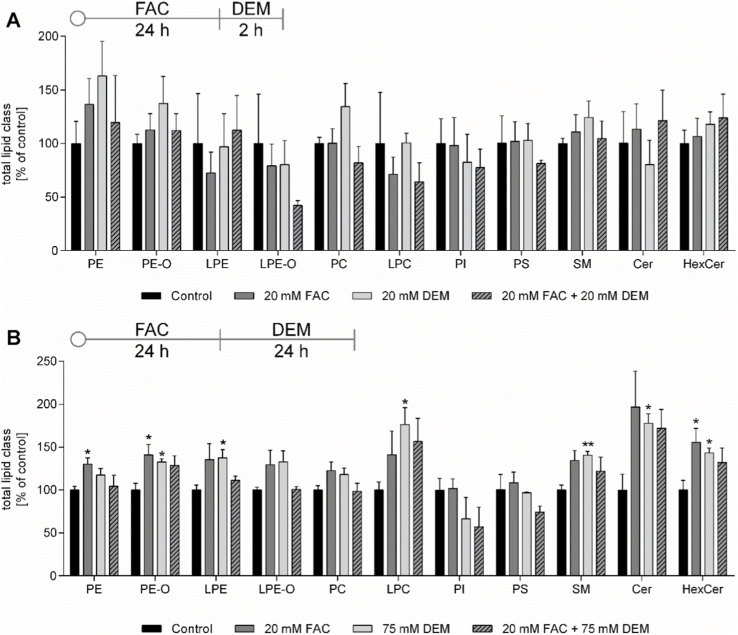
Relative distribution of phospho- (PL) and sphingolipid (SL) subclasses determined from the peak areas of LC-MS analysis. Measurement was conducted after treatment with FAC and 2 h (A) or 24 h (B) DEM and areas were normalized to protein content and untreated control. Shown are the mean + SEM of ≥ 3 independent experiments. Significance is depicted as ∗ compared to untreated control. Abbreviations: PE phosphatidylethanolamine; PE-O plasmanyl-phosphatidylethanolamine; LPE lysophosphatidylethanolamine; PC phosphatidylcholine; LPC lysophosphatidylcholine; PI phosphatidylinositol; PS phosphatidylserine; SM sphingomyelin; Cer ceramide; HexCer hexosylceramide.

**Fig. 8:**
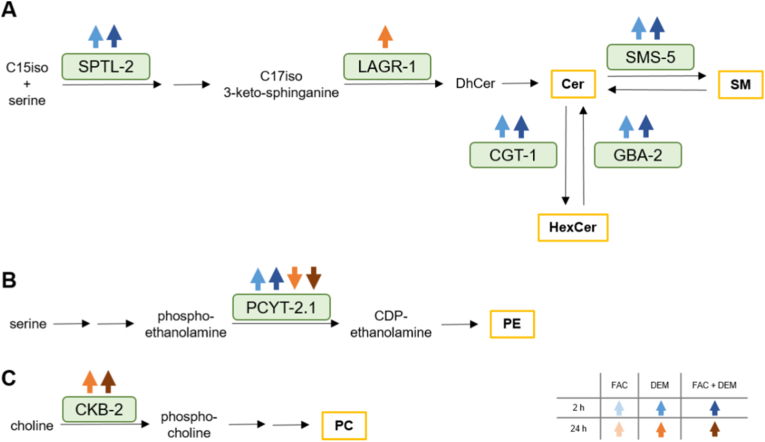
Proteins encoded by DEGs after treatment with FAC and DEM from the biosynthetic pathways of A) Ceramide (Cer), hexosylceramide (HexCer), and sphingomyelin (SM), B) phosphatidylethanolamine (PE), and C) phosphatidylcholine (PC). Transcriptomic analysis was performed from n = 3 independent experiments. *C. elegans*/human orthologue: SPTL-2/SPTLC-2: serine palmitoyl transferase; LAGR-1/CERS-1: sphingosine N-acyltransferase; SMS-5/SMS-1: sphingomyelin synthase; CGT-1/UGCG: ceramide glucosyltransferase; GBA-2/GBA-1: glucosylceramidase; PCYT-2.1/PCYT-2: ethanolamine-phosphate cytidylyltransferase; CKB-2/CHKB: choline kinase.

In addition to CL, numerous PL and SL are part of cellular and cell organelle membranes, as well as involved in several signal transduction processes related to stress response, cell death, and neuronal function. The distributions of the 9 PL and 3 SL subclasses, measured in untreated control at both treatment times, are shown in Fig. S7. While no changes in total content of the different lipid classes could be observed after 2 h DEM treatment (Fig. 7A), FAC, DEM, and the combined treatment after 24 h led to a similar extent to increased levels of certain PL and SL subclasses (Fig. 7B). After the long-term treatment, phosphatidylethanolamine (PE), plasmanyl-PE (PE-O), lyso-PE (LPE), lysophosphatidylcholine (LPC), sphingomyelin (SM), ceramide (Cer), and hexosyl-Cer (HexCer) were affected. In addition, treatment with FAC alone and in combination with DEM led to alterations in the distribution of the number of double bonds in PE and PC (Fig. S8). However, no changes in MDA content, which is a byproduct of lipid peroxidation, could be measured in our study (Fig. S9). The transcriptomic analysis revealed that several genes associated with the biosynthesis of Cer, HexCer, SM, PE, and PC were affected by DEM treatment, particularly after 2 h (Fig. 8).

### 3.6 FAC and DEM affected neuronal endpoints

**Fig. 9:**
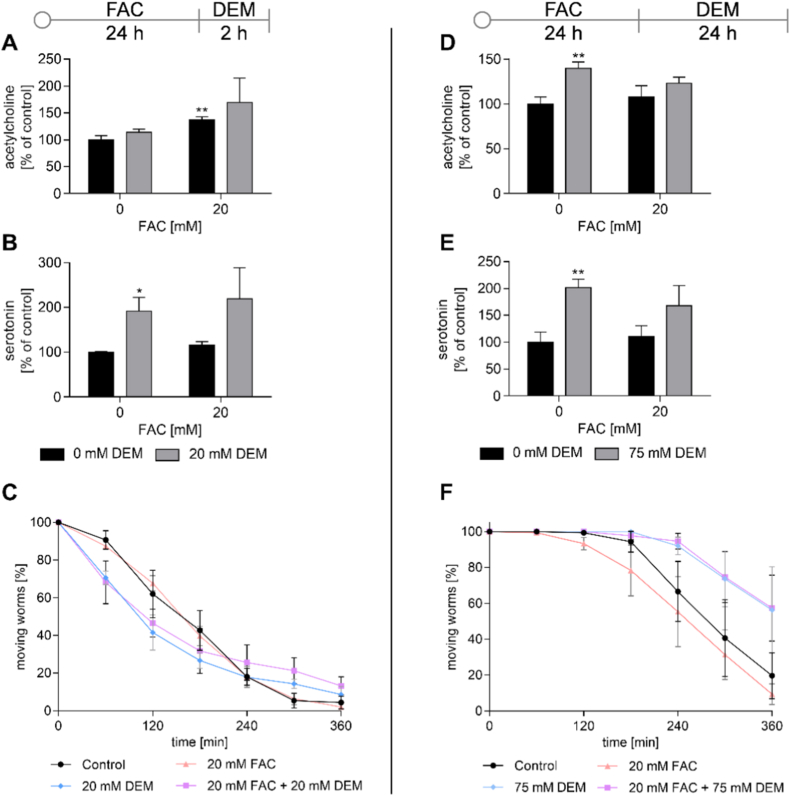
Neurotransmitter levels and moving worm fraction of the aldicarb-sensitivity assay after treatment with FAC and 2 h (A–C) or 24 h (D–F) treatment with DEM. A, D) Acetylcholine and B, E) serotonin levels were measured via HPLC-MS/MS and normalized to internal standard, protein content, and untreated control. C, F) Fraction of moving worms after treatment with aldicarb. Shown are the mean + SEM of ≥ 3 independent experiments. Significance is depicted as ∗ compared to untreated control.

Since neurons require a lot of energy and are therefore rich in mitochondria, we investigated whether FAC and DEM affect neurotransmitter levels. Treatment with FAC led to an increase in acetylcholine levels (Fig. 9A), which were recovered to control levels 24 h post FAC treatment (Fig. 9D). While short-term treatment with DEM led to an increase in serotonin levels (Fig. 9B), both serotonin and acetylcholine levels were elevated after long-term treatment (Fig. 9D and E). None of the exposure conditions affected dopamine and γ-aminobutyric acid levels (Fig. S10).

**Fig. 10:**
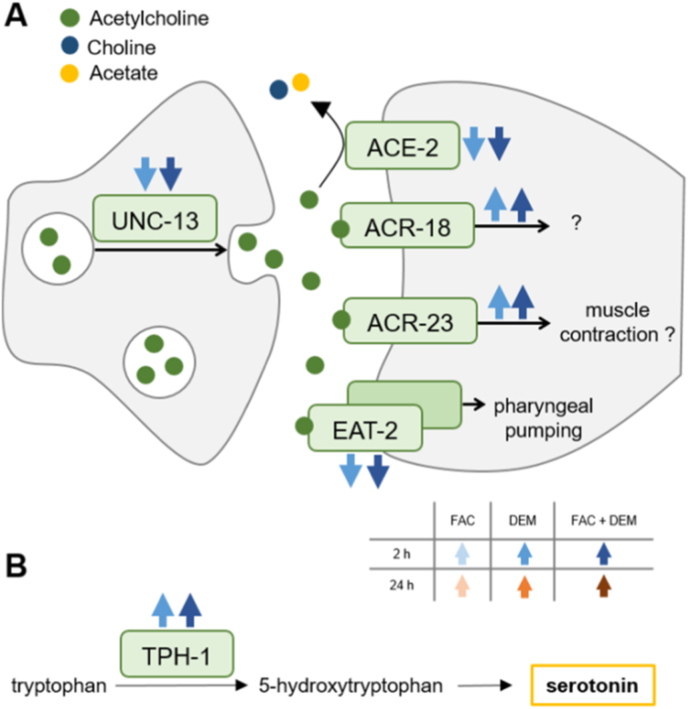
Proteins encoded by DEGs after short-term treatment with DEM and in combination with FAC of A) acetylcholine and B) serotonin-associated genes. Transcriptomic analysis was performed from n = 3 independent experiments each. *C. elegans*/human orthologue: UNC-13/UNC-13; ACE-2/BCHE: acetylcholinesterase; ACR-18/23: acetylcholine receptor; EAT-2/CHRNA-1: subunit of nicotinic acetylcholine receptor; TPH-1/TPH-1/2: tryptophan hydroxylase.

Since FAC and DEM affected acetylcholine levels, we investigated whether the synaptic transmission rate of acetylcholine in the neuromuscular junction was also altered. Therefore, we examined the sensitivity to the acetylcholinesterase inhibitor aldicarb, which leads to paralysis of the worms when applied over a longer period [40]. The short-term treatment with DEM led to a slightly earlier paralysis of the worms compared to untreated control (Fig. 9C), but this may also be due to the lower survival rate (Fig. 4A), as no distinction could be made between paralyzed and dead worms. Following long-term treatment with DEM, a trend for resistance to aldicarb could be observed, which would indicate a lower transmission rate of acetylcholine compared to untreated control (Fig. 9F). Treatment with FAC alone did not lead to any changes in aldicarb sensitivity. Short-term treatment with DEM alone and in combination with FAC led to DEGs of acetylcholinesterase, acetylcholine receptors, and serotonin biosynthesis compared to untreated control (Fig. 10).

## 4 Discussion and conclusions

Fe is essential for various biological processes, but in terms of overdosage it is also discussed in context of several diseases like neurodegenerative diseases, particularly those linked to aging. This could be associated with age-related weakening of various cellular systems, such as antioxidant defense [44]. However, there is a lack of studies addressing effects of Fe overdosage and a decreased antioxidant capacity particularly in terms of affected mechanisms and potential counter regulating effects in a living organism.

First of all, we were able to confirm that FAC treatment led to elevated Fe levels in *C. elegans* and we identified adverse effects upon the treatment. By treating L4 worms with 20 mM FAC for 24 h, we could gain a fivefold enrichment of total Fe content compared to untreated control. Although the difference was smaller 24 h post FAC treatment, it was still threefold higher compared to control, which is probably due to the lack of an active excretion mechanism of Fe [45]. Valentini et al. treated *C. elegans* eggs until young adults with 9 mM FAC and measured a twofold increase of free Fe(III) using electron paramagnetic resonance. The treatment had no effect on lifespan, but increased *ftn-1::gfp* expression and led to decreased resistance to *tert*-butyl hydroperoxide [46]. James et al. achieved approximately 50 % increased Fe levels after 48 h treatment with 5 mg/mL FAC in young adult worms and showed that *ftn-2* plays a dominant role in Fe storage in *C. elegans*. FAC treatment resulted in increased FTN-2 stored Fe in this study and led to a reduced lifespan in *ftn-2* null worms [47,48]. The differential expression of *ftn-1* and the unchanged Fe(II) content after FAC treatment in our study suggest that the high amounts of Fe were stored at least to a large extent as Fe(III) in this storage protein, which can contain up to 1500 Fe atoms in *C. elegans* [49]. Even though no altered gene expression of *ftn-2* was measured in transcriptomic and RT-qPCR analysis, FTN-2 is also expected to be involved in storing the excess Fe in our study. PCA analysis shows little variance between the FAC treated samples and the untreated controls. This suggests that FAC exposure is well buffered and needs only minor changes in gene expression. However, qPCR measurements show an upregulation of *fpn-1.2* expression after FAC treatment, which could indicate that Fe should be exported more efficiently. Nevertheless, 24 h post FAC treatment, nematodes showed significantly increased PE and PE-O amounts and trends toward higher amounts of SM, Cer, and HexCer. These two PL subclasses form the largest lipid fraction in the cell membrane and are therefore important for its function and integrity, whereby ether lipids in particular are essential for the fluidity and function of membrane proteins [50]. The three SL subclasses are also located in the cell membrane and are found primarily in lipid rafts, where they are essential for the function of receptors and signaling proteins [51]. The composition of lipids is therefore of great importance, and changes are discussed in the context of neurodegenerative diseases such as AD. While several studies have measured decreased PE levels in the brains of AD patients, some studies have found increased SM and Cer levels [52]. Wang et al. showed effects of FAC on lipid metabolism in *C. elegans*, as FAC treatment led to increased fatty acid uptake by the fatty acid transmembrane transporter ACS-20 and lipid accumulation [53]. Total Fe accumulation is also discussed in the context of AD, where aging becomes a risk factor, as Fe homeostasis is no longer well-regulated [54]. Furthermore, FAC treatment led to a slight increase in the neurotransmitter acetylcholine, which was at the same level as the untreated control 24 h post FAC treatment. Studies in rat brains and *in vitro* indicate that Fe treatment can inhibit acetylcholinesterase activity, which could lead to an increase of the neurotransmitter [55,56]. In our study, however, FAC does not affect the transmission rate of acetylcholine in neuromuscular junction, as no difference between FAC-treated and untreated nematodes could be observed in the behavioral assay with aldicarb. In addition to its function as an excitatory neurotransmitter in important processes such as muscle contraction, acetylcholine is also discussed as a neuromodulator [57].

We could further identify DEM as a suitable substance to model a lower antioxidant capacity in nematodes. With the short-term DEM treatment for 2 h, we decreased the GSH level with 20 mM DEM to 30 % compared to untreated control. Although no alterations could be measured after long-term treatment for 24 h, the GSH-DEM product was still detectable in the samples, as with short-term treatment. The fact that there are numerous GST genes among the DEGs after both treatment times indicates that they are involved in the binding of DEM to GSH in *C. elegans*, which has also been shown *in vitro* by Kubal et al. [15]. The DEGs showed after both treatment times upregulated genes of GSH synthesis. This may indicate an increase in GSH production, which could lead to no difference in GSH level between long-term DEM treatment and untreated control due to counter regulation. While short-term treatment with DEM led to a twofold increase in Fe(II) content, this effect could not be observed after long-term treatment, when GSH levels were similar to untreated control. Jenkins et al. also showed an increase of Fe(II) in *C. elegans* by treatment with DEM for 6 h and described a buffer limit of the organism when 30 % of the Fe is present as Fe(II), while the Fe(II) content was 41 % higher than in untreated control [16]. DEM treatment also led to increased expression of *fpn-1.2* after 2 h, and of both *fpn-1.1* and *fpn-1.2* after 24 h, highlighting the impact of DEM on Fe homeostasis. The GSH depletion not only promotes the presence of reactive Fe-species but also impairs the antioxidant defense, with cells less capable of repairing damage caused by RONS. The fact that two out of eight GPX orthologues were upregulated after short-term DEM treatment could also be due to increased reactive Fe(II) species at this time point, as these may lead to lipid peroxidation [10]. Although we could not measure alterations in the content of the lipid peroxidation byproduct MDA, there were changes in the total contents of PE-O, LPE, LPC, SM, Cer, and HexCer, especially after long-term treatment with DEM. Transcriptomic analysis revealed that both DEM treatment scenarios led to DEGs in the biosynthesis of PE, PC, SM, Cer, and HexCer. Additionally, we detected a slight decrease in CL content, which may be associated with slightly lower mitochondrial masses after DEM treatment. Since up to 15 % of cellular GSH is found in the mitochondria and these organelles are exposed to the constant formation of RONS, GSH depletion could lead to mitochondrial impairment and thus to mitophagy [58]. In our study, short-term treatment with DEM showed a slight trend toward lower gene expression of mitochondrial expressed *nd-1*, whereas long-term treatment resulted in increased levels. This suggests that DEM had an impact on mitochondrial transcriptional activity [59]. Cornell et al. showed that the mitochondrial stress response in the form of unfolded protein response (UPR^mt^) in *C. elegans* is coordinated by the neurotransmitters γ-aminobutyric acid and acetylcholine signaling. They showed that UPR^mt^ induction, measured as HSP-6 expression, is associated with the lack of acetylcholinesterase and with elevated systemic acetylcholine levels [43]. In our study, short-term treatment with DEM led not only to decreased mitochondrial masses but also to upregulation of *hsp-6*, while long-term treatment resulted in elevated acetylcholine levels and altered aldicarb sensitivity. Additionally, Cornell et al. showed that the lack of the acetylcholinesterases *ace-1* and *ace-2* increased mitochondrial fragmentation, and in our study, short-term treatment with DEM led to lower expression of the *ace-2* gene [43].

Interestingly, the combined treatment, which was associated with a combination of low GSH and increased total Fe and Fe(II) levels, did not lead to significant effects compared to DEM or FAC alone for any of the endpoints investigated in this study. One reason for this could be sufficient regulation of Fe homeostasis during FAC treatment, whereby the excess Fe is stored in the organism in a largely redox-inert form.

**Fig. 11:**
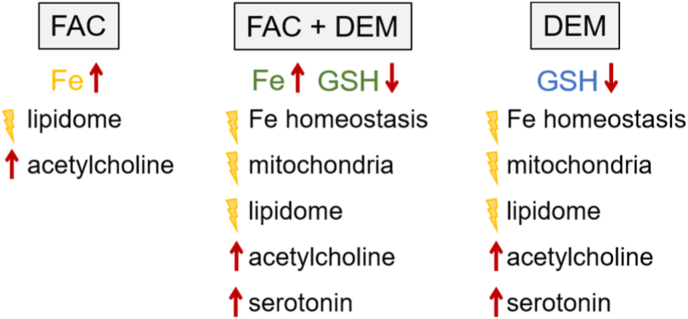
Schematic overview of the effects of FAC and/or DEM treatment.

To sum up, *C. elegans* treatment with FAC affects the acetylcholine level as well as PL and SL distributions even after prolonged time without further Fe treatment (Fig. 11). In addition, it could be shown that increased total Fe levels do not necessarily lead to increased reactive Fe(II) species, but short-term treatment with DEM and presumably GSH depletion increased Fe(II) species and affected genes associated with Fe homeostasis in *C. elegans*. Furthermore, short-term treatment with DEM led to lower mitochondrial masses, increased gene expression of *hsp-6*, increased serotonin level, and differentially expressed genes associated with PL, SL, acetylcholine, and serotonin homeostasis. The consequences of long-term treatment with DEM were lower mitochondrial mass, increased PL, SL, acetylcholine, and serotonin levels, and differentially expressed genes associated with PL and SL synthesis. Overall, we were able to show that *C. elegans* is a suitable organism to model Fe overdosage by FAC and lower antioxidant capacity by DEM treatment. The two DEM treatment times indicate acute, compensatory response, and long-lasting effects. Even if the long-term DEM treatment is not reducing GSH levels due to counter regulation we could identify long-lasting adverse consequences for the nematodes.

## Funding

This project was supported by the 10.13039/501100001659DFG Research Unit TraceAge (FOR 2558, BO4103/4-2) and by the 10.13039/501100001659DFG Research Infrastructure NGS CC (project 407495230) as part of the Next Generation Sequencing Competence Network (project 423957469). NGS analyses were carried out at the Competence Centre for Genomic Analysis (Kiel).

## CRediT authorship contribution statement

**Anna Gremme:** Conceptualization, Formal analysis, Investigation, Methodology, Visualization, Writing – original draft, Writing – review & editing. **Emely Gerisch:** Investigation, Writing – review & editing. **Dominik Wieland:** Investigation, Methodology, Writing – review & editing. **Julia Hillebrand:** Investigation, Writing – review & editing. **Franziska Drews:** Formal analysis, Writing – review & editing. **Marcello Pirritano:** Formal analysis, Writing – review & editing. **Ann-Kathrin Weishaupt:** Methodology, Writing – review & editing. **Janina Fuss:** Investigation. **Vera Schwantes:** Investigation, Methodology. **Johannes Scholz:** Writing – review & editing. **Vivien Michaelis:** Methodology, Writing – review & editing. **Alicia Thiel:** Methodology, Writing – review & editing. **Gawain McColl:** Writing – review & editing. **Bernhard Michalke:** Writing – review & editing. **Martin Simon:** Writing – review & editing. **Heiko Hayen:** Supervision, Writing – review & editing. **Julia Bornhorst:** Conceptualization, Funding acquisition, Investigation, Methodology, Project administration, Writing – review & editing.

## Declaration of competing interest

The authors declare that they have no known competing financial interests or personal relationships that could have appeared to influence the work reported in this paper.

## Data Availability

Data will be made available on request.

## References

[1] Beard J.L. (2001). Iron biology in immune function, muscle metabolism and neuronal functioning. J. Nutr..

[2] Puig S., Ramos-Alonso L., Romero A.M., Martínez-Pastor M.T. (2017). The elemental role of iron in DNA synthesis and repair. Metallomics.

[3] Winterbourn C.C. (1995). Toxicity of iron and hydrogen peroxide: the fenton reaction. Toxicol. Lett..

[4] Fraga C.G., Oteiza P.I. (2002). Iron toxicity and antioxidant nutrients. Toxicology.

[5] Dev S., Babitt J.L. (2017). Overview of iron metabolism in health and disease, hemodialysis international. International Symposium on Home Hemodialysis.

[6] Ghadery C., Pirpamer L., Hofer E., Langkammer C., Petrovic K., Loitfelder M., Schwingenschuh P., Seiler S., Duering M., Jouvent E., Schmidt H., Fazekas F., Mangin J.-F., Chabriat H., Dichgans M., Ropele S., Schmidt R. (2015). R2∗ mapping for brain iron: associations with cognition in normal aging. Neurobiol. Aging.

[7] Turck D., Bohn T., Castenmiller J., de Henauw S., Hirsch-Ernst K.-I., Knutsen H.K., Maciuk A., Mangelsdorf I., McArdle H.J., Pentieva K., Siani A., Thies F., Tsabouri S., Vinceti M., Aggett P., Fairweather-Tait S., de Sesmaisons Lecarré A., Fabiani L., Karavasiloglou N., Saad R.M., Sofroniou A., Titz A., Naska A., EFSA Panel on Nutrition, Novel Foods and Food Allergens (2024). Scientific opinion on the tolerable upper intake level for iron. EFSA J..

[8] Detcheverry F., Senthil S., Narayanan S., Badhwar A. (2023). Changes in levels of the antioxidant glutathione in brain and blood across the age span of healthy adults: a systematic review. Neuroimage, Clin..

[9] Deponte M. (2013). Glutathione catalysis and the reaction mechanisms of glutathione-dependent enzymes. Biochim. Biophys. Acta Gen. Subj..

[10] Conrad M., Kagan V.E., Bayir H., Pagnussat G.C., Head B., Traber M.G., Stockwell B.R. (2018). Regulation of lipid peroxidation and ferroptosis in diverse species. Gene Dev..

[11] Hider R.C., Kong X.L. (2011). Glutathione: a key component of the cytoplasmic labile iron pool. Biometals an international journal on the role of metal ions in biology, biochemistry, and medicine.

[12] Lill R., Hoffmann B., Molik S., Pierik A.J., Rietzschel N., Stehling O., Uzarska M.A., Webert H., Wilbrecht C., Mühlenhoff U. (2012). The role of mitochondria in cellular iron–sulfur protein biogenesis and iron metabolism. Biochim. Biophys. Acta Mol. Cell Res..

[13] Federico A., Cardaioli E., Da Pozzo P., Formichi P., Gallus G.N., Radi E. (2012). Mitochondria, oxidative stress and neurodegeneration. J. Neurol. Sci..

[14] Cheng R., Dhorajia V.V., Kim J., Kim Y. (2022). Mitochondrial iron metabolism and neurodegenerative diseases. Neurotoxicology.

[15] Kubal G., Meyer D.J., Norman R.E., Sadler P.J. (1995). Investigations of glutathione conjugation in vitro by 1H NMR spectroscopy. Uncatalyzed and glutathione transferase-catalyzed reactions. Chem. Res. Toxicol..

[16] Jenkins N.L., James S.A., Salim A., Sumardy F., Speed T.P., Conrad M., Des Richardson R., Bush A.I., McColl G., Gruber J., Tyler J.K. (2020). Changes in ferrous iron and glutathione promote ferroptosis and frailty in aging Caenorhabditis elegans. eLife.

[17] Gremme A., Al-Timimi Safa Flaih Z., Scholz J., Gerisch E., Thiel A., McColl G., Hayen H., Michalke B., Bornhorst J. (2025). Is ferric the same as ferrous? Effect of nutritionally relevant iron species in C. Elegans: bioavailability, iron homeostasis, oxidative stress, and cell death. J. Agric. Food Chem..

[18] Kubens L., Weishaupt A.-K., Michaelis V., Rohn I., Mohr F., Bornhorst J. (2024). Exposure to the environmentally relevant fungicide maneb: studying toxicity in the soil nematode Caenorhabditis elegans. Environ. Int..

[19] Brenner S. (1974). The genetics of Caenorhabditis elegans. Genetics.

[20] Scholz J., Rudt E., Gremme A., Gaßmöller C.M., Bornhorst J., Hayen H. (2024). Hyphenation of supercritical fluid chromatography and trapped ion mobility-mass spectrometry for quantitative lipidomics. Anal. Chim. Acta.

[21] Thiel A., Weishaupt A.-K., Nicolai M.M., Lossow K., Kipp A.P., Schwerdtle T., Bornhorst J. (2023). Simultaneous quantitation of oxidized and reduced glutathione via LC-MS/MS to study the redox state and drug-mediated modulation in cells, worms and animal tissue. J. Chromatogr., B: Anal. Technol. Biomed. Life Sci..

[22] Bornhorst J., Chakraborty S., Meyer S., Lohren H., Brinkhaus S.G., Knight A.L., Caldwell K.A., Caldwell G.A., Karst U., Schwerdtle T., Bowman A., Aschner M. (2014). The effects of pdr1, djr1.1 and pink1 loss in manganese-induced toxicity and the role of α-synuclein in C. elegans. Metallomics integrated biometal science.

[23] Martin M. (2011). Cutadapt removes adapter sequences from high-throughput sequencing reads, EMBnet. journal.

[24] Love M.I., Huber W., Anders S. (2014). Moderated estimation of fold change and dispersion for RNA-Seq data with DESeq2. Genome Biol..

[25] Ashburner M., Ball C.A., Blake J.A., Botstein D., Butler H., Cherry J.M., Davis A.P., Dolinski K., Dwight S.S., Eppig J.T., Harris M.A., Hill D.P., Issel-Tarver L., Kasarskis A., Lewis S., Matese J.C., Richardson J.E., Ringwald M., Rubin G.M., Sherlock G. (2000). Gene ontology: tool for the unification of biology. Nat. Genet..

[26] Aleksander S.A., Balhoff J., Carbon S., Cherry J.M., Drabkin H.J., Ebert D., Feuermann M., Gaudet P., Harris N.L., Hill D.P., Lee R., Mi H., Moxon S., Mungall C.J., Muruganugan A., Mushayahama T., Sternberg P.W., Thomas P.D., van Auken K., Ramsey J., Siegele D.A., Chisholm R.L., Fey P., Aspromonte M.C., Nugnes M.V., Quaglia F., Tosatto S., Giglio M., Nadendla S., Antonazzo G., Attrill H., dos Santos G., Marygold S., Strelets V., Tabone C.J., Thurmond J., Zhou P., Ahmed S.H., Asanitthong P., Luna Buitrago D., Erdol M.N., Gage M.C., Ali Kadhum M., Li K.Y.C., Long M., Michalak A., Pesala A., Pritazahra A., Saverimuttu S.C.C., Su R., Thurlow K.E., Lovering R.C., Logie C., Oliferenko S., Blake J., Christie K., Corbani L., Dolan M.E., Ni L., Sitnikov D., Smith C., Cuzick A., Seager J., Cooper L., Elser J., Jaiswal P., Gupta P., Naithani S., Lera-Ramirez M., Rutherford K., Wood V., de Pons J.L., Dwinell M.R., Hayman G.T., Kaldunski M.L., Kwitek A.E., Laulederkind S.J.F., Tutaj M.A., Vedi M., Wang S.-J., D'Eustachio P., Aimo L., Axelsen K., Bridge A., Hyka-Nouspikel N., Morgat A., Engel S.R., Karra K., Miyasato S.R., Nash R.S., Skrzypek M.S., Weng S., Wong E.D., Bakker E., Berardini T.Z., Reiser L., Auchincloss A., Argoud-Puy G., Blatter M.-C., Boutet E., Breuza L., Casals-Casas C., Coudert E., Estreicher A., Livia Famiglietti M., Gos A., Gruaz-Gumowski N., Hulo C., Jungo F., Le Mercier P., Lieberherr D., Masson P., Pedruzzi I., Pourcel L., Poux S., Rivoire C., Sundaram S., Bateman A., Bowler-Barnett E., Bye-A-Jee H., Denny P., Ignatchenko A., Ishtiaq R., Lock A., Lussi Y., Magrane M., Martin M.J., Orchard S., Raposo P., Speretta E., Tyagi N., Warner K., Zaru R., Diehl A.D., Chan J., Diamantakis S., Raciti D., Zarowiecki M., Fisher M., James-Zorn C., Ponferrada V., Zorn A., Ramachandran S., Ruzicka L., Westerfield M., The Gene Ontology Consortium (2023). The gene ontology knowledgebase in 2023. Genetics.

[27] Thomas P.D., Ebert D., Muruganujan A., Mushayahama T., Albou L.-P., Mi H. (2022). PANTHER: making genome-scale phylogenetics accessible to all. Protein science a publication of the Protein Society.

[28] Bonnot T., Gillard M., Nagel D. (2019). A simple protocol for informative visualization of enriched gene ontology terms. Bio-protocol.

[29] Rooney J.P., Ryde I.T., Sanders L.H., Howlett E.H., Colton M.D., Germ K.E., Mayer G.D., Greenamyre J.T., Meyer J.N. (2015). PCR based determination of mitochondrial DNA copy number in multiple species. Methods Mol. Biol..

[30] Baesler J., Michaelis V., Stiboller M., Haase H., Aschner M., Schwerdtle T., Sturzenbaum S.R., Bornhorst J. (2021). Nutritive manganese and zinc overdosing in aging C. elegans result in a metallothionein-mediated alteration in metal homeostasis. Mol. Nutr. Food Res..

[31] Livak K.J., Schmittgen T.D. (2001). Analysis of relative gene expression data using real-time quantitative PCR and the 2(-Delta Delta C(T)) method. Methods (San Diego, Calif.).

[32] Neumann C., Baesler J., Steffen G., Nicolai M.M., Zubel T., Aschner M., Bürkle A., Mangerich A., Schwerdtle T., Bornhorst J. (2020). The role of poly(ADP-ribose) polymerases in manganese exposed Caenorhabditis elegans. Journal of trace elements in medicine and biology organ of the Society for Minerals and Trace Elements (GMS).

[33] Morton K.S., Wahl A.K., Meyer J.N. (2024). The effect of common paralytic agents used for fluorescence imaging on redox tone and ATP levels in Caenorhabditis elegans. PLoS One.

[34] Bornhorst J., Ebert F., Lohren H., Humpf H.-U., Karst U., Schwerdtle T. (2012). Effects of manganese and arsenic species on the level of energy related nucleotides in human cells. Metallomics.

[35] Matyash V., Liebisch G., Kurzchalia T.V., Shevchenko A., Schwudke D. (2008). Lipid extraction by methyl-tert-butyl ether for high-throughput lipidomics. Journal of Lipid Research.

[36] Helmer P.O., Nicolai M.M., Schwantes V., Bornhorst J., Hayen H. (2021). Investigation of cardiolipin oxidation products as a new endpoint for oxidative stress in C. elegans by means of online two-dimensional liquid chromatography and high-resolution mass spectrometry. Free Radic. Biol. Med..

[37] Schmid R., Heuckeroth S., Korf A., Smirnov A., Myers O., Dyrlund T.S., Bushuiev R., Murray K.J., Hoffmann N., Lu M., Sarvepalli A., Zhang Z., Fleischauer M., Dührkop K., Wesner M., Hoogstra S.J., Rudt E., Mokshyna O., Brungs C., Ponomarov K., Mutabdžija L., Damiani T., Pudney C.J., Earll M., Helmer P.O., Fallon T.R., Schulze T., Rivas-Ubach A., Bilbao A., Richter H., Nothias L.-F., Wang M., Orešič M., Weng J.-K., Böcker S., Jeibmann A., Hayen H., Karst U., Dorrestein P.C., Petras D., Du X., Pluskal T. (2023). Integrative analysis of multimodal mass spectrometry data in MZmine 3. Nat. Biotechnol..

[38] Weishaupt A.-K., Gremme A., Meiners T., Schwantes V., Sarnow K., Thiel A., Schwerdtle T., Aschner M., Hayen H., Bornhorst J. (2024). Dysfunctional copper homeostasis in Caenorhabditis elegans affects genomic and neuronal stability. Redox Biochemistry and Chemistry.

[39] Weishaupt A.-K., Kubens L., Ruecker L., Schwerdtle T., Aschner M., Bornhorst J. (2023). A reliable method based on liquid chromatography-tandem mass spectrometry for the simultaneous quantification of neurotransmitters in Caenorhabditis elegans. Molecules (Basel, Switzerland).

[40] Mahoney T.R., Luo S., Nonet M.L. (2006). Analysis of synaptic transmission in Caenorhabditis elegans using an aldicarb-sensitivity assay. Nat. Protoc..

[41] Hirayama T., Niwa M., Hirosawa S., Nagasawa H. (2020). High-throughput screening for the discovery of iron homeostasis modulators using an extremely sensitive fluorescent probe. ACS Sens..

[42] Presley A.D., Fuller K.M., Arriaga E.A. (2003). MitoTracker green labeling of mitochondrial proteins and their subsequent analysis by capillary electrophoresis with laser-induced fluorescence detection. J. Chromatogr. B.

[43] Cornell R., Cao W., Harradine B., Godini R., Handley A., Pocock R. (2024). Neuro-intestinal acetylcholine signalling regulates the mitochondrial stress response in Caenorhabditis elegans. Nat. Commun..

[44] Emir U.E., Raatz S., McPherson S., Hodges J.S., Torkelson C., Tawfik P., White T., Terpstra M. (2011). Noninvasive quantification of ascorbate and glutathione concentration in the elderly human brain. NMR Biomed..

[45] Kohgo Y., Ikuta K., Ohtake T., Torimoto Y., Kato J. (2008). Body iron metabolism and pathophysiology of iron overload. Int. J. Hematol..

[46] Valentini S., Cabreiro F., Ackerman D., Alam M.M., Kunze M.B.A., Kay C.W.M., Gems D. (2012). Manipulation of in vivo iron levels can alter resistance to oxidative stress without affecting ageing in the nematode C. elegans. Mechanisms of ageing and development.

[47] James S.A., Roberts B.R., Hare D.J., de Jonge M.D., Birchall I.E., Jenkins N.L., Cherny R.A., Bush A.I., McColl G. (2015). Direct in vivo imaging of ferrous iron dyshomeostasis in ageing Caenorhabditis elegans. Chem. Sci..

[48] James S.A., Hare D.J., Jenkins N.L., de Jonge M.D., Bush A.I., McColl G. (2016). φXANES: in vivo imaging of metal-protein coordination environments. Sci. Rep..

[49] Mubarak S.S.M., Malcolm T.R., Brown H.G., Hanssen E., Maher M.J., McColl G., Jameson G.N.L. (2023). Biochemical characterization of Caenorhabditis elegans ferritins. Biochemistry.

[50] Koyiloth M., Gummadi S.N. (2022). Regulation and functions of membrane lipids: insights from Caenorhabditis elegans. BBA advances.

[51] Luo H., Zhao X., Wang Z.-D., Wu G., Xia Y., Dong M.-Q., Ma Y. (2024). Sphingolipid profiling reveals differential functions of sphingolipid biosynthesis isozymes of Caenorhabditis elegans. Journal of Lipid Research.

[52] Kosicek M., Hecimovic S. (2013). Phospholipids and alzheimer's disease: alterations, mechanisms and potential biomarkers. Int. J. Mol. Sci..

[53] Wang H., Jiang X., Wu J., Zhang L., Huang J., Zhang Y., Zou X., Liang B. (2016). Iron overload coordinately promotes ferritin expression and fat accumulation in Caenorhabditis elegans. Genetics.

[54] Tian Y., Tian Y., Yuan Z., Zeng Y., Wang S., Fan X., Yang D., Yang M. (2022). Iron metabolism in aging and age-related diseases. Int. J. Mol. Sci..

[55] Perez V.P., Lima de, Martins Maria Noêmia, Da Silva R.S., Dornelles A.S., Vedana G., Bogo M.R., Bonan C.D., Schröder N. (2010). Iron leads to memory impairment that is associated with a decrease in acetylcholinesterase pathways. Curr. Neurovascular Res..

[56] Pohanka M. (2014). Copper, aluminum, iron and calcium inhibit human acetylcholinesterase in vitro. Environ. Toxicol. Pharmacol..

[57] Picciotto M.R., Higley M.J., Mineur Y.S. (2012). Acetylcholine as a neuromodulator: cholinergic signaling shapes nervous system function and behavior. Neuron.

[58] Santacroce G., Gentile A., Soriano S., Novelli A., Lenti M.V., Di Sabatino A. (2023). Glutathione: pharmacological aspects and implications for clinical use in non-alcoholic fatty liver disease. Front. Med..

[59] Castellani C.A., Longchamps R.J., Sun J., Guallar E., Arking D.E. (2020). Thinking outside the nucleus: mitochondrial DNA copy number in health and disease. Mitochondrion.

