# Supplementary material for: Consequences of iron exposure and glutathione depletion on redox balance, lipidome, and neurotransmission in C. elegans
Source: Redox Biol. 2026 Jan 12;90:104023. doi: 10.1016/j.redox.2026.104023 (PMC12856550; doi:10.1016/j.redox.2026.104023)
Supplement: Multimedia component 1 [file mmc1.docx]

**Supporting Information**

**Consequences of Iron Exposure and Glutathione Depletion on Redox Balance, Lipidome, and Neurotransmission in *C. elegans***

Anna Gremme^1^, Emely Gerisch^1^, Dominik Wieland^2^, Julia Hillebrand^2^, Franziska Drews^3^, Marcello Pirritano^3^, Ann-Kathrin Weishaupt^1^, Janina Fuss^4^, Vera Schwantes^2^, Johannes Scholz^2^, Vivien Michaelis^1^, Alicia Thiel^1^, Gawain McColl^5^, Bernhard Michalke^6^, Martin Simon^3^, Heiko Hayen^2^, Julia Bornhorst^1,7*^

^1^Food Chemistry with focus on toxicology, Faculty of Mathematics and Natural Science, University of Wuppertal, 42119 Wuppertal, Germany

^2^Institute of Inorganic and Analytical Chemistry, University of Münster, 48149 Münster, Germany

^3^Molecular Cell Biology and Microbiology, Faculty of Mathematics and Natural Science, University of Wuppertal, 42119 Wuppertal, Germany

^4^Competence Centre for Genomic Analysis, CCGA, 24118 Kiel, Germany

^5^Florey Institute of Neuroscience and Mental Health, The University of Melbourne, Parkville Victoria 3052, Australia

^6^Chemconsulting, 85570 Markt Schwaben, Germany

^7^TraceAge - DFG Research Unit on Interactions of Essential Trace Elements on Healthy and Diseased Elderly (FOR 2558), Berlin-Potsdam-Jena-Wuppertal, Germany

**Correspondence:** Prof. Dr. Julia Bornhorst, Food Chemistry with Focus on Toxicology, Faculty of Mathematics and Natural Sciences, University of Wuppertal, Gaußstr. 20, 42119 Wuppertal, Germany; E-mail: bornhorst@uni-wuppertal.de

**S1. Method parameters for GSH-DEM determination**

To identify the product GSH-DEM and its fragments by LC-MS/MS, the reaction of GSH and DEM was carried out *in vitro* based on Kubal et al. *^1^* For the stock solutions, DEM was diluted in DMSO and GSH was weighed in freshly and dissolved in 0.2 M phosphate buffer. The reaction was performed at equimolar amounts of both reagents (5 mM) and 1% DMSO in phosphate buffer for 3.5 h at 20 °C. Figure S1 shows the structure of GSH-DEM with the fragmentation patterns measured in this study. The used collision energy, collision cell exit potential, and declustering potential are shown in Table S1. Figure S2 shows representative chromatograms of the synthesized GSH-DEM standard (Fig. S2A) and a *C. elegans* sample after 2 h DEM treatment (Fig S2B).


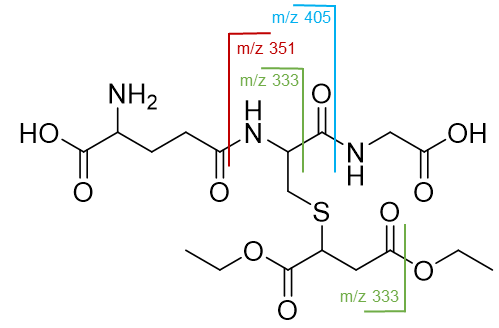


**Fig. S1.** Structural formula of the GSH-DEM product with the fragmentation patterns and the corresponding m/z.

**Table S1.** Parameters for detection of the fragments of GSH-DEM. Quantifier is marked with an asterisk.

| **Fragmentation**  **[m/z]** | **Collision energy**  **[V]** | **Collision cell exit potential [V]** | **Declustering potential [V]** |
| --- | --- | --- | --- |
| 480 > 333 | 23 | 10 | 25 |
| 480 > 351* | 21 | 10 | 25 |
| 480 > 405 | 26 | 10 | 25 |





**Fig. S2.** Representative MRM chromatograms of A) mass transitions of GSH-DEM in phosphate buffer and B) GSSG, GSH, and GSH-DEM in *C. elegans* treated with 20 mM DEM for 2 h.

**S2. Chemicals**

The isotopically labeled lipid standard 1,2-dipalmitoyl-d62-*sn*-glycero-3-phosphocholine (PC 32:0-d62 [PC 16:0 / 16:0 - d62]) was obtained from Biomol GmbH (Hamburg, Germany, PN 28751). 16:1 cardiolipin (CL 64:4), ammonium formate (pur. ≥99.995%) and ammonium acetate (≥99.99%) were purchased from Sigma-Aldrich Corporation (Steinheim, Germany). Butylhydroxytoluene was acquired from Fisher Scientific GmbH (Schwerte, Germany). LC grade chloroform and methyl-*tert*-butylether (MTBE) were obtained from Merck KGaA (Darmstadt, Germany). LC-MS grade acetonitrile (ACN), methanol (MeOH) and 2-propanol (IPA) as well as acetic (≥99.8%) and formic acid (≥99%) were obtained from VWR International GmbH (Darmstadt, Germany). Water was purified utilizing a Milli-Q EQ 7000 system purchased from Merck KGaA (Molsheim, France).

**S3. Lipid extraction**

For lipid extraction of *C*. *elegans* samples (4500 adult day 1 and 2 nematodes each), an extraction according to the protocol by Matyash *et al*. was performed as follows.*^2,3^* The pellets were resuspended with 200 µL of water and three freeze-thaw cycles utilizing liquid nitrogen were applied. Afterwards, 20 µL of a 40 µM solution of PC 32:0-d62 was added prior to extraction. The solution was adapted to a final concentration of 2 µM for phospholipid (PL) and sphingolipid (SL) determination. This isotopically labeled internal standard was utilized to ensure proper extraction. Additionally, 20 µL of a 65 mM butylhydroxytoluene solution as antioxidant were added. Before sonication in an ultrasonic processor (20 min, P = 20 W, C = 50 %, A = 80 %; UP200St, Hielscher Ultrasonic GmbH, Germany), 750 µL of ice cold MeOH were added. Subsequently, 750 µL of MeOH and 5 mL of MTBE were added and samples incubated for 1 h under agitation (700 rpm, MULTI-TX5, VELP Scientifica Srl, Italy) at room temperature. Furthermore, 1.25 mL of water were added and samples were incubated for 15 min. Phase separation was supported by centrifugation at 5000 rpm for 5 min (Centrifuge 5804, Eppendorf AG, Germany). After collection of the upper organic phase, another extraction cycle of the remaining aqueous phase was performed by adding 2 mL of MTBE/MeOH/Water (10/3/2.5, *v*/*v*). Combined organic phases were evaporated under a gentle nitrogen stream at 40°C. The residue was reconstituted in 1000 µL of MTBE/MeOH (3/1, *v*/*v*) and subsequently aliquoted for the analysis of cardiolipins (800 µL) as well as PL and SL (200 µL). For PL and SL determination, 200 µL of sample extract were dried under a gentle nitrogen stream at 40°C and the residue reconstituted with 80 µL of IPA. For cardiolipin analysis all 800 µL of sample extract was resuspended in 80 µL of IPA containing CL 64:4 (0.5 M), after drying under a gentle nitrogen stream at 40°C.

**S4. Lipid Nomenclature**

Lipid denomination is based on the shorthand notation established by Liebisch *et al*.*^4,5^* Phospholipids are described by their respective subclass notation, followed by the total number of carbon atoms (X) and double bonds (Y) found in the fatty acyl chains, e.g. PC X:Y. For sphingolipid species the total number of carbon atoms (X) and double bonds (Y) between the sphingoid base and fatty acyl chain are given, similar to phospholipid nomenclature. Additionally, the number of hydroxy groups (Z) is given, e.g. SM X:Y;OZ.

**S5. Chromatographic setup for determination of CL composition**

For 2D chromatographic separation of CL, a Vanquish Flex Duo UHPLC-system (Thermo Scientific, Dionex, Dreieich, Germany; Dual Split Sampler FT, Dual Pump F, Column Compartments H) incorporating a 6-port valve was utilized. Xcalibur 4.1 software and the SII Chromeleon plugin were used for instrument operation. Phospholipid classes were separated in the first dimension using an iHILIC Fusion(+) column (250 x 2.1 mm, 3.5 μm, 100 Å, HILICON AB, Umeå, Sweden) and a gradient based on our previous work, comprising an ammonium formate buffer (20 mM, pH 3.5, 5% ACN, A1) and ACN (B1).*^3^* Before a series of measurements, a 20-minute equilibration step at 60% B1 was employed to attain a reproducible HILIC separation and retention times. Additional chromatographic parameters included a flow rate of 0.4 mL/min, an injection volume of 2 µL and a column temperature maintained at 40°C. The HILIC gradient was initiated at 95% B1 for two minutes, followed by a linear decrease to 60% B1 within 15 min. This mobile phase composition was maintained for six minutes before being increased to 95% B1 where it was held for the remainder of the method (33 min total run time). Intra-class separation of CL species in the second chromatographic dimension was achieved utilizing a RP-HPLC XSelect Premier CSH C18 column (100 x 2.1 mm, 2.5 μm, 100 Å; Waters Corporation, Milford, MA, USA) under gradient elution. The gradient system was composed of an aqueous ammonium acetate buffer (10 mM, pH 3.5, 5% MeOH) (A2) and MeOH/IPA (60/40, *v*/*v*, containing 10 mM ammonium acetate buffer, 0.01% acetic acid; B2). Further HPLC parameters included a flow rate of 0.3 mL/min at 40°C column temperature. The RP-HPLC gradient started at 80% B2 and was held for 14 min, then linearly increased to 92% B2 in one minute. Subsequently, B2 was further increased to 98% over 14 min, followed by an increase to 100% B2 within 0.5 min. After maintaining this composition for 2.5 min, B2 was decreased to 80% within 0.4 min and held until the end of the run. In the 2D-LC heart-cut setup, both chromatographic dimensions were connected through a 6-port valve and a valve configuration as previously described.*^3^* The timing of the 6-port valve switching was determined by the elution window of the CL-class (heart-cut window: 12.1-12.7 min). To enable separation in the second dimension, the heart-cut fraction was collected in a 500 µL sample loop.

**S6. Mass spectrometric setup for determination of CL composition**

For CL analysis via 2D-HPLC heart-cut MS/MS, mass spectrometric detection was carried out using a Q Exactive Plus mass spectrometer and a heated electrospray ionization source (HESI-II, Thermo Scientific, Bremen, Germany). Electrospray ionization was conducted in negative ionization mode, and HESI-II probe parameters were set as follows: source voltage -3.5 kV, probe heater temperature 300 °C, sheath gas flow rate 45 arbitrary units, auxiliary gas flow rate 10 arbitrary units, spare gas flow rate 1 arbitrary unit, and capillary temperature 325 °C. The s-lens rf level was set to 85. Full MS measurements were performed with a resolution of 140,000 (FWHM at *m/z* 200), an AGC target set to 3e^6^ and a maximum C-trap injection time of 100 ms. For structural elucidation, data-dependent MS/MS acquisitions were obtained at a normalized collision energy of 24 eV (based on a *m*/*z* of 500) and a resolution of 17.500 (at *m*/*z* 200). The isolation window for precursors was set to 1.5 Da. For MS/MS experiments, a maximum C-trap injection time of 50 ms was applied.

**S7. Chromatographic setup for determination of PL and SL composition**

Chromatographic separation of phospholipid (PL) and sphingolipid (SL) species was performed utilizing an UltiMate3000 UHPLC system (Thermo Fisher Scientific GmbH, Dreieich, Germany) consisting of the following modules. A DGP-3600RS dual gradient pump module, WPS-3000TRS autosampler, SRD-3600 degasser and TCC-3000SD column oven. RP separation was carried out with an XSelect Premier CSH C18 column (100 x 2.1 mm, 2.5 μm, 100 Å; Waters Corporation, Milford, MA, USA). With this, a binary gradient of A: H_2_O/MeOH (95/5, *v*/*v*) and B: IPA/MeOH (85/15, *v*/*v*) with a total runtime of 28.5 min was applied as depicted in Table S2. A constant flow rate of 0.3 mL/min, column oven temperature of 40°C and injection volume of 3 µL were maintained.

**Table S2:** Binary gradient for RP separation of phospholipids with A: H_2_O/MeOH (95/5, *v*/*v*) and B: IPA/MeOH (85/15, *v*/*v*).

| time [min] | 0.0 | 1.0 | 2.0 | 16.0 | 18.0 | 24.0 | 24.5 | 28.5 |
| --- | --- | --- | --- | --- | --- | --- | --- | --- |
| % B | 60 | 60 | 72 | 80 | 100 | 100 | 60 | 60 |

**S8. Mass spectrometric setup for determination of PL and SL composition**

Mass spectrometric measurements for PL and SL determination including ion mobility spectrometry were carried out on a timsTOF fleX mass spectrometer (Bruker Daltonics GmbH, Bremen, Germany). Parameters for electrospray ionization (ESI) -trapped ion mobility spectrometry (TIMS) -tandem mass spectrometry (MS/MS) were adapted from Rudt *et al*. and tailored to the here required conditions.*^6^*

All measurements were performed in negative ionization mode with these following ESI source settings: end plate offset 500 V, capillary voltage 3500 V, nebulizer pressure 2.0 bar, dry gas flow rate 9.0 l/min, and dry gas temperature 220 °C.

For ion mobility spectrometry measurements via TIMS, a mobility range of 0.8 – 1.65 Vs/cm^2^ in combination with a ramp time of 300 ms was applied. The utilized tunnel voltages were set as following: Δt1 20.0 V, Δt2 120.0 V, Δt3 -80.0 V, Δt4 -100.0 V, Δt5 0.0 V, Δt6 -100.0 V, collision cell in -220.0 V. Additionally the accumulation was locked to the mobility range and ion accumulation was limited to a target value of 7.5 million via ion charge control for reduction of overcharge effects.

A mass range of *m*/*z* 100 – 1350 combined with the following ion transfer parameters was applied: deflection 1 delta -80.0 V, funnel 1 RF 360.0 Vpp, isCID energy -0.0 eV, funnel 2 RF 250.0 Vpp, multipole RF 200.0 Vpp. Collision cell energy and RF were set to 10.0 eV and 1100.0 Vpp respectively. Quadrupole ion energy and low mass settings were 5.0 eV and *m*/*z* 150.0. Transfer time and pre pulse storage for pre TOF focus were set to 65.0 µs and 7.0 µs respectively.

For MS/MS experiments, data dependent acquisition (dda) with parallel accumulation serial fragmentation (PASEF) was utilized. Fragmentation via dda-PASEF was applied with a collision energy of 40 eV for ions between a charge range of 0 – 1 and a *m*/*z* range of 300 – 1350. Quadrupole ion selection was based on an isolation width of *m*/*z* 1.75 with an intensity threshold of 100 and a target intensity of 4000. Selected ions were actively excluded and released from selection after 0.1 min. For each cycle two PASEF ramps were applied.

Initial mass calibration was performed according to sodium formate clusters utilizing a 5 mM solution of sodium formate in IPA/H_2_O (1/1, *v*/*v*). For initial ion mobility calibration, a tuning mix (ESI-L Low Concentration Tuning Mix; Agilent Technologies Inc., Santa Clara, CA, USA) was used with calibration points depicted in Table S3. Additionally, an online calibration has been applied after each measurement utilizing a mixed mass and mobility calibrant solution (1/1, *v*/*v*). Therefore a 20 µL sample loop was connected to the six-port valve of the timsTOF fleX instrument and continuously filled with calibrant solution via the integrated syringe pump and a flow rate of 1 µL/min. Valve switching at minute 26.5 of the LC run introduced the calibrant into the timsTOF fleX instrument via LC flow.

**Table S3:** Calibration for mass spectrometric measurements utilizing a 5 mM sodium formate solution (left) and mobility calibration with ESI-L Low Concentration Tuning Mix (right).

| **Calibration point** | **Mass Calibration** | **Mobility Calibration** | |
| --- | --- | --- | --- |
|  | *m*/*z* | *m*/*z* | Mobility [V/cm^2^] |
| 1 | 112.9856 | 601.9790 | 0.8824 |
| 2 | 180.9731 | 1033.9881 | 1.2582 |
| 3 | 248.9605 | 1333.9689 | 1.4073 |
| 4 | 316.9479 |  |  |
| 5 | 384.9353 |  |  |
| 6 | 452.9227 |  |  |
| 7 | 520.9102 |  |  |
| 8 | 588.8976 |  |  |
| 9 | 656.885 |  |  |
| 10 | 724.8724 |  |  |
| 11 | 792.8599 |  |  |
| 12 | 860.8473 |  |  |
| 13 | 928.8347 |  |  |
| 14 | 996.8221 |  |  |
| 15 | 1064.8096 |  |  |
| 16 | 1132.797 |  |  |
| 17 | 1200.7844 |  |  |
| 18 | 1268.7718 |  |  |
| 19 | 1336.7593 |  |  |

**S9. Data Processing and Lipid Identification for determination of CL composition**

Chromatographic data processing and identification of CLs were performed using the open-source software MZmine 4 (version 4.2.0; mzio GmbH, Bremen, Germany).*^7^* The annotation of CL species was based on accurate mass and matching MS/MS spectra. A batch processing method was created for negative ionization mode with noise levels set at 5000 for MS1 and 250 for MS2, respectively. For the ADAP chromatogram builder a mass tolerance of 15 ppm was set.

**S10. Data Processing and Lipid Identification for determination of PL and SL composition**

The data processing, annotation and identification workflow was performed utilizing Metaboscape 2023b software (Bruker Daltonics GmbH, Bremen, Germany). For data processing the following parameters were applied. Features were extracted between a retention time of 0.5 – 24.5 min and a *m*/*z* of 100 – 1350. Furthermore, a minimum 4D peak size of 50 data points, recursive feature extraction of 25 data points and intensity threshold of 200 counts was required. Primary ions were selected as [M-H]^-^ while seed ions were [M+CH_3_COO]^-^, [M+Cl]^-^, [M-2H]^2-^ and common ions were [M-H-H_2_O]^-^ all with a 0.8 EIC correlation. For annotation of extracted features, the integrated rule-based lipid annotation as well as external MSDIAL-TandemMassSpectralAtlas-VS68-Neg library were applied.*^8,9^* Respective narrow and wide tolerances were set as CCS 2% and 5%, *m*/*z* 2.0 ppm and 5.0 ppm, MS/MS score 800 and 500, 85 mSigma (isotopic pattern matching). Annotated features were revised manually utilizing lipid-class specific 4D-Kendrick mass plots (KMD (CH_2_), *m*/*z*, *t*_R_, CCS) and considering deviation values calculated by Metaboscape software.*^10^*





**Fig. S3.** Zn levels after treatment with FAC and 2 h (A) or 24 h (B) DEM. Shown are mean + SEM of ≥ 3 independent experiments. Significance is depicted as * compared to untreated control and # to DEM treatment only.

**
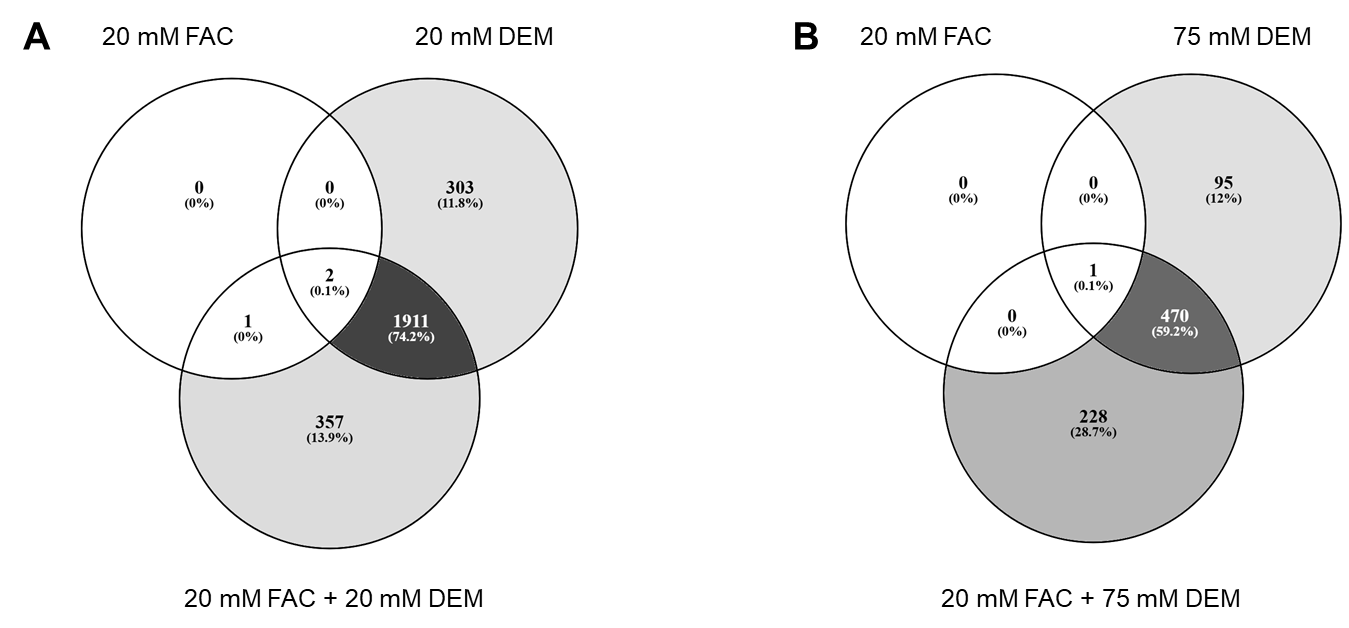
**

**Fig. S4.** Number of up- and down-regulated DEGs after treatment with FAC and 2 h (A) and 24 h (B) DEM. Visualized using Venny.*^11^*

**Table S2.** List of GO terms with corresponding GO numbers.

| GO name | GO number |
| --- | --- |
| ABC-type transporter activity | GO:0140359 |
| acyl-CoA hydrolase activity | GO:0016289 |
| alcohol dehydrogenase (NAD+) activity | GO:0004022 |
| alcohol dehydrogenase [NADP)+] activity | GO:0018455 |
| alcohol dehydrogenase [NADP+] activity | GO:0018455 |
| binding | GO:0005488 |
| catalytic activity | GO:0003824 |
| cation binding | GO:0043169 |
| DNA-binding transcription factor activity | GO:0003700 |
| fatty acid elongase activity | GO:0009922 |
| flavin adenine dinucleotide binding | GO:0050660 |
| FMN binding | GO:0010181 |
| G protein-coupled receptor activity | GO:0004930 |
| galactoside binding | GO:0016936 |
| glucuronosyltransferase activity | GO:0015020 |
| glutathione disulfide oxidoreductase activity | GO:0015038 |
| glutathione transferase activity | GO:0004364 |
| glycosyltransferase activity | GO:0016757 |
| heme binding | GO:0020037 |
| hexose transmembrane transporter activity | GO:0015149 |
| hexosyltransferase activity | GO:0016758 |
| hypotaurine monooxygenase activity | GO:0047822 |
| ion binding | GO:0043167 |
| iron ion binding | GO:0005506 |

Continuation of Table S2.

| GO name | GO number |
| --- | --- |
| lysozyme activity | GO:0003796 |
| metal ion binding | GO:0046872 |
| molecular function | GO:0003674 |
| molecular transducer activity | GO:0060089 |
| monooxygenase activity | GO:0004497 |
| mRNA 5'-diphosphatase activity | GO:0034353 |
| N,N-dimethylaniline monooxygenase activity | GO:0004499 |
| NAD(P)H oxidase H2O2-forming activity | GO:0016174 |
| oxidoreductasae activity | GO:0016491 |
| oxidoreductasae activity, acting on CH-OH group of donors | GO:0016614 |
| oxidoreductase activity, acting on NAD(P)H, oxygen as acceptor | GO:0050664 |
| oxidoreductase activty, acting on paired donors, with incorporation or reduction of molecular oxygen, reduced flavin or flavoprotein as one donor, and incorporation of one atom of oxygen | GO:0016712 |
| oxidoreductase activity, acting on the CH-OH group of donors, NAD or NADP as acceptor | GO:0016616 |
| sequence-specific DNA binding | GO:0043565 |
| signaling receptor activity | GO:0038023 |
| small molecule binding | GO:0036094 |
| sodium ion transmembrane transporter activity | GO:0015081 |
| structural constituent of chromatin | GO:0030527 |
| tetrapyrrole binding | GO:0046906 |
| transcription regulator activity | GO:0140110 |
| transferase activity | GO:0016740 |
| transferase activity, transferring alkyl or aryl (other than methyl) groups | GO:0016765 |
| transition metal ion binding | GO:0046914 |
| transmembrane transporter activity | GO:0022857 |
| transporter activity | GO:0005215 |
| UDP-glycosyltransferase activity | GO:0008194 |
| zinc ion binding | GO:0008270 |





**Fig. S5.** Cellular energy charge value after treatment with FAC and 2h (A) or 24 h (B) DEM. Shown are mean + SEM of ≥ 3 independent experiments. Significance is depicted as * compared to untreated control. C+: 1 h treatment with 100 µM sodium azide as positive control.





**Fig. S6.** Distribution of CL species after treatment with FAC and 2 h (A) or 24 h (B) DEM. Shown are mean + SEM of ≥ 3 (2h DEM n = 2) independent experiments. Significance is depicted as * compared to untreated control.





**Fig. S7.** Distribution of phospho- and sphingolipids determined from peak areas in d1 adult (A) and d2 adult (B) untreated controls. Distribution of phospho- and sphingolipids based on the degree of saturation after treatment with FAC and 2 h (C) or 24 h (D) DEM. Shown are mean + SEM of ≥ 3 independent experiments.





**Fig. S8.** Distribution of PE (A, B) and PC (C, D) based on the degree of saturation after treatment with FAC and 2 h (A, C) or 24 h (B, D) DEM. Shown are mean + SEM of ≥ 3 independent experiments. Significance is depicted as * compared to untreated control.





**Fig. S9.** Total MDA content after treatment with FAC and 2 h (A) or 24 h (B) DEM. Shown are mean + SEM of ≥ 3 independent experiments.





**Fig. S10.** Dopamine (A, C) and γ-aminobutyric acid (B, D) content normalized to protein amount and untreated control after treatment with FAC and 2 h (A, B) or 24 h (C, D) DEM. Shown are mean + SEM of ≥ 3 independent experiments.

**References**

(1) Kubal, G.; Meyer, D. J.; Norman, R. E.; Sadler, P. J. Investigations of glutathione conjugation in vitro by 1H NMR spectroscopy. Uncatalyzed and glutathione transferase-catalyzed reactions. *Chemical research in toxicology* **1995**, *8*, 780–791.

(2) Matyash, V.; Liebisch, G.; Kurzchalia, T. V.; Shevchenko, A.; Schwudke, D. Lipid extraction by methyl-tert-butyl ether for high-throughput lipidomics. *Journal of Lipid Research* **2008**, *49*, 1137–1146.

(3) Helmer, P. O.; Nicolai, M. M.; Schwantes, V.; Bornhorst, J.; Hayen, H. Investigation of cardiolipin oxidation products as a new endpoint for oxidative stress in C. elegans by means of online two-dimensional liquid chromatography and high-resolution mass spectrometry. *Free Radical Biology and Medicine* **2021**, *162*, 216–224.

(4) Liebisch, G.; Vizcaíno, J. A.; Köfeler, H.; Trötzmüller, M.; Griffiths, W. J.; Schmitz, G.; Spener, F.; Wakelam, M. J. Shorthand notation for lipid structures derived from mass spectrometry. *Journal of Lipid Research* **2013**, *54*, 1523–1530.

(5) Liebisch, G.; Fahy, E.; Aoki, J.; Dennis, E. A.; Durand, T.; Ejsing, C. S.; Fedorova, M.; Feussner, I.; Griffiths, W. J.; Köfeler, H.; Merrill, A. H., JR; Murphy, R. C.; O'Donnell, V. B.; Oskolkova, O.; Subramaniam, S.; Wakelam, M. J. O.; Spener, F. Update on LIPID MAPS classification, nomenclature, and shorthand notation for MS-derived lipid structures. *Journal of Lipid Research* **2020**, *61*, 1539–1555.

(6) Rudt, E.; Feldhaus, M.; Margraf, C. G.; Schlehuber, S.; Schubert, A.; Heuckeroth, S.; Karst, U.; Jeck, V.; Meyer, S. W.; Korf, A.; Hayen, H. Comparison of Data-Dependent Acquisition, Data-Independent Acquisition, and Parallel Reaction Monitoring in Trapped Ion Mobility Spectrometry-Time-of-Flight Tandem Mass Spectrometry-Based Lipidomics. *Analytical Chemistry* **2023**, *95*, 9488–9496.

(7) Schmid, R.; Heuckeroth, S.; Korf, A.; Smirnov, A.; Myers, O.; Dyrlund, T. S.; Bushuiev, R.; Murray, K. J.; Hoffmann, N.; Lu, M.; Sarvepalli, A.; Zhang, Z.; Fleischauer, M.; Dührkop, K.; Wesner, M.; Hoogstra, S. J.; Rudt, E.; Mokshyna, O.; Brungs, C.; Ponomarov, K.; Mutabdžija, L.; Damiani, T.; Pudney, C. J.; Earll, M.; Helmer, P. O.; Fallon, T. R.; Schulze, T.; Rivas-Ubach, A.; Bilbao, A.; Richter, H.; Nothias, L.-F.; Wang, M.; Orešič, M.; Weng, J.-K.; Böcker, S.; Jeibmann, A.; Hayen, H.; Karst, U.; Dorrestein, P. C.; Petras, D.; Du, X.; Pluskal, T. Integrative analysis of multimodal mass spectrometry data in MZmine 3. *Nature biotechnology* **2023**, *41*, 447–449.

(8) Kind, T.; Liu, K.-H.; Lee, D. Y.; DeFelice, B.; Meissen, J. K.; Fiehn, O. LipidBlast in silico tandem mass spectrometry database for lipid identification. *Nature methods* **2013**, *10*, 755–758.

(9) Tsugawa, H.; Ikeda, K.; Takahashi, M.; Satoh, A.; Mori, Y.; Uchino, H.; Okahashi, N.; Yamada, Y.; Tada, I.; Bonini, P.; Higashi, Y.; Okazaki, Y.; Zhou, Z.; Zhu, Z.-J.; Koelmel, J.; Cajka, T.; Fiehn, O.; Saito, K.; Arita, M.; Arita, M. A lipidome atlas in MS-DIAL 4. *Nature biotechnology* **2020**, *38*, 1159–1163.

(10) Korf, A.; Vosse, C.; Schmid, R.; Helmer, P. O.; Jeck, V.; Hayen, H. Three-dimensional Kendrick mass plots as a tool for graphical lipid identification. *Rapid communications in mass spectrometry RCM* **2018**, *32*, 981–991.

(11) Oliveros, J. C. Venny. An interactive tool for comparing lists with Venn's diagrams. *https://bioinfogp.cnb.csic.es/tools/venny/index.html* **2007 - 2015**.
